# An efficient protocol for extracting thylakoid membranes and total leaf proteins from *Posidonia oceanica* and other polyphenol-rich plants

**DOI:** 10.1186/s13007-024-01166-7

**Published:** 2024-03-11

**Authors:** Quentin Charras, Pascal Rey, Dorian Guillemain, Fabian Dourguin, Hugo Laganier, Sacha Peschoux, Roland Molinié, Marwa Ismaël, Stefano Caffarri, Catherine Rayon, Colette Jungas

**Affiliations:** 1 CEA, CNRS, BIAM, LGBP Team, Aix-Marseille University, Marseille, France; 2 Science for Life Laboratory, School of Engineering Sciences in Chemistry, Biotechnology and Health, KTH-Royal Institute of Technology, KTH University, Stockholm, Sweden; 3 CEA, CNRS, BIAM, P&E Team, Aix-Marseille University, Saint Paul-Lez-Durance, France; 4 CNRS, IRD, IRSTEA, OSU Institut Pythéas, Aix-Marseille University, Marseille, France; 5 UFR Informatique, mathématiques et mathématiques appliquées (IM2AG), Université Grenoble Alpes, Saint Martin d’Heres, France; 6 UMR INRAE 1158 Transfrontalière BioEcoAgro, BIOlogie des Plantes et Innovation (BIOPI), UPJV, Amiens, France

**Keywords:** Seagrasses, Wild plants, Thylakoid membranes, Polyphenols, Ascorbic acid (vitamin C), Polyethylene glycol, Photosystems, Native-PAGE, SDS-PAGE

## Abstract

**Background:**

The extraction of thylakoids is an essential step in studying the structure of photosynthetic complexes and several other aspects of the photosynthetic process in plants. Conventional protocols have been developed for selected land plants grown in controlled conditions. Plants accumulate defensive chemical compounds such as polyphenols to cope with environmental stresses. When the polyphenol levels are high, their oxidation and cross-linking properties prevent thylakoid extraction.

**Results:**

In this study, we developed a method to counteract the hindering effects of polyphenols by modifying the grinding buffer with the addition of both vitamin C (VitC) and polyethylene glycol (PEG4000). This protocol was first applied to the marine plant *Posidonia oceanica* and then extended to other plants synthesizing substantial amounts of polyphenols, such as *Quercus pubescens* (oak) and *Vitis vinifera* (grapevine). Native gel analysis showed that photosynthetic complexes (PSII, PSI, and LHCII) can be extracted from purified membranes and fractionated comparably to those extracted from the model plant *Arabidopsis thaliana.* Moreover, total protein extraction from frozen *P. oceanica* leaves was also efficiently carried out using a denaturing buffer containing PEG and VitC.

**Conclusions:**

Our work shows that the use of PEG and VitC significantly improves the isolation of native thylakoids, native photosynthetic complexes, and total proteins from plants containing high amounts of polyphenols and thus enables studies on photosynthesis in various plant species grown in natural conditions.

**Supplementary Information:**

The online version contains supplementary material available at 10.1186/s13007-024-01166-7.

## Background

The ecological crisis is accelerating the need to study photosynthesis in free-growing wild plants to complement data obtained on plants grown under controlled laboratory conditions. Indeed, the latter conditions do not consider the ecological and evolutionary relevance of the underlying photosynthetic mechanisms. For example, studies of marine plants rarely address the response mechanisms to a light environment that strongly differ depending on the depth, at which the plants live (1–40 m). One of the most important molecular approaches for studying photosynthesis is the biochemical characterization of the photosystems II and I (PSII and PSI). Extraction of thylakoid membranes and photosynthetic protein complexes is crucial to performing such studies. However, thylakoid extraction protocols have been optimized for model plants grown in laboratory conditions, such as *Arabidopsis thaliana* or *Pisum sativum* [1–3], and proved very inefficient in extracting membranes from wild plants and crops like oak and grapevine. Protocols that extract proteins by denaturing them directly from leaves are available for some refractory species. However, those protocols require protein precipitation in organic buffers such as acetone, acetone/trichloroacetic acid, or phenol solvents followed by several washing and drying steps [4–7]. They have the additional inconvenience of being time-consuming due to the need for multiple steps.

*Posidonia oceanica*, an endemic Mediterranean seagrass is the engineer of an ecosystem of major importance that provides goods and services to the growing human populations in coastal areas. Initial attempts to extract thylakoids and proteins from leaves of *P. oceanica* with the conventional protocol used for model plants failed. Thylakoid preparations turned brown and resisted solubilization by non-ionic detergents, likely due to the presence of polyphenols [8–10]. Plants in natural conditions are subjected to continuous changes in environmental factors such as light quantity and quality, temperature, UV radiation, and water availability. Many plant species synthesize specialized secondary metabolic compounds like polyphenols [11]. These organic compounds are characterized by the presence of several hydroxyl groups linked to an aromatic ring, which confer protective redox and reactive properties upon abiotic and biotic stress [12–14]. The hydroxyl groups connected through a C–C delocalized double bond promote the oxidation of oxygen atoms within the molecule by molecular oxygen or reactive oxygen species. In this reaction, polyphenols turn into quinones, which display highly oxidizing properties in vitro [8, 10, 15]. Those molecules also possess in vitro cross-linking and reticulating properties mediated by hydrogen bonds between their hydroxyl and carbonyl groups [14, 16–18].

In plants, polyphenols are compartmentalized within the cell vacuole, but also in specialized polyphenol-rich cells [19–21]. Grinding leaves during the thylakoid membrane extraction releases polyphenols and allow them to interact with oxygen leading to harmful effects on macromolecules.

In this study, we developed a method to isolate thylakoid membranes and total proteins from the leaves of *P. oceanica*, a polyphenol-rich plant. We showed that, by neutralizing the reactivity of polyphenols using both VitC and PEG, we can easily and efficiently extract thylakoid proteins from *P. oceanica*. The addition of PEG and VitC in the denaturing buffer enhances the total protein extraction from leaves. These compounds were previously used to extract thylakoids from *Picea abies* [22], but no explanation was given for their use in the extraction buffer and no relationship was established with the plant's polyphenol content. Our method extended to other plant species enriched in phenols or in polyphenols: *Quercus pubescens* (oak), *Vitis vinifera* (grapevine), and *Ocimum basilicum* (basil) [23, 24]. In *Q. pubescens* and *V. vinifera*, using the new optimized protocol allows the efficient isolation of thylakoids compared to conventional protocols.

## Results

### Extraction of thylakoids from *P. oceanica* leave*s* using a conventional procedure

**Fig. 1:**
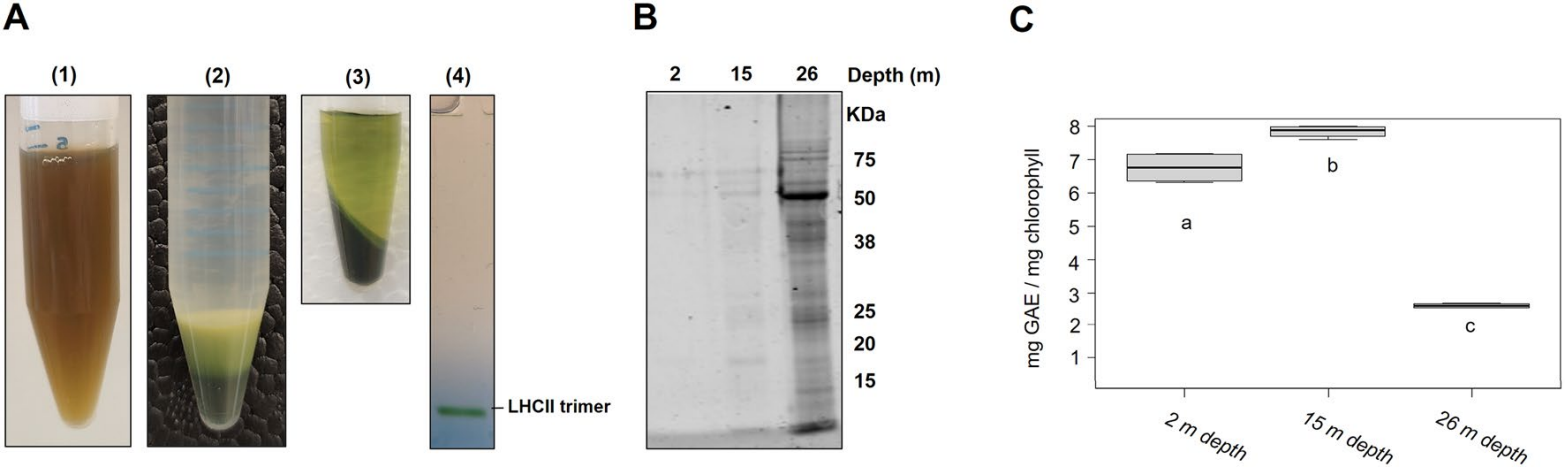
Conventional procedure results in inefficient thylakoid and protein extraction from *P. oceanica* leaves. **A** Thylakoids were extracted from *P. oceanica* leaves harvested at a 2 m depth using the conventional protocol, without PEG and VitC. (1) Sample of the filtered ground leaf mixture. (2) Final pellet after resuspending the storage buffer. (3) Centrifuged pellet of the extracted thylakoids solubilized in 2% α-DDM per mg/mL of chlorophyll (20mg DDM for 1mg chlorophyll). (4) The protein content of the solubilizate analyzed by BN-PAGE. **B** Coomassie-blue-stained SDS-PAGE of total protein samples extracted from *P. oceanica* leaves from three different growing depths using conventional Laemmli denaturing buffer. Extraction was carried out using equal amounts of leaf material and equal volumes of protein extracts were loaded on the gels. **C** The concentration of soluble polyphenols in *P. oceanica* leaves from the three different growing depths per leaf chlorophyll. *GAE* gallic acid equivalent. Data are represented as the median, quartiles, and border values of 4 technical replicates. Different letters indicate significant differences at *P* < 0.05, as determined using the Shapiro–Wilk normality test followed by the Student’s t-distribution analysis. Data and calculations are presented in Additional file 1: Data S1

When *P. oceanica* (from 2-m depth) leaves were ground according to the conventional protocol (i.e. without Asc and PEG,), the resulting mixture appeared brownish (Fig. 1A left). In addition, the pellet obtained at the last centrifugation step (cf Method section for details) displayed membranes that appeared entrapped in a mucilaginous mass (Fig. 1A middle). The brownish color of the supernatant indicated the possible formation of quinones as a result of polyphenol oxidation during grinding [8, 9, 15] and the mucilaginous mass was likely a consequence of both the presence of cross-linked quinones and polyphenols. The final pellet described above was then resuspended in a solubilization buffer for Native-PAGE analysis. Solubilization of thylakoids by dodecyl maltoside detergent (DDM) was ineffective as seen by the clear color of the supernatant after solubilization (Fig. 1A top right). The separation of the solubilized sample in the BN-PAGE revealed only free LHCII trimers.

### Extraction of total proteins from *P. oceanica* leaves

Extracting total proteins from *P. oceanica* leaves was tested to prepare soluble and membrane protein fractions for SDS-PAGE and immunoblot analyses. Similar amounts of fresh Posidonia leaves were used for protein extraction, (*ca* 1 g) in 2 mL of buffer. To evaluate the relative quantity and quality of the extracted proteins, we performed SDS-PAGE gels. First, a typical Tris extraction buffer containing, sodium dodecyl sulfate (SDS), a reducing agent (β-mercaptoethanol), and a protease inhibitor (PMSF) was used. That protocol is efficient for preparing protein extracts from glycophytes such as *Arabidopsis thaliana* [25], *Solanum tuberosum* [26], and halophytes such as *Thellungiella halophila* [27] or *Atriplex halimus* [28]. As shown in Fig. 1B, after separating proteins by SDS-PAGE and Coomassie-blue staining, little protein was recovered from samples collected at 2 m depth. A similar profile was observed in the sample harvested at 15 m depth. Intriguingly, more proteins were recovered from samples at 26 m depth although the bands appeared quite smeary, likely due to a distinct composition of *P. oceanica* leaves in this environment. These data indicate that the solubilization problem persists even under denaturing conditions and that a strong ionic detergent such as SDS is not enough to inhibit the probable deleterious effects of polyphenols. To test whether the presence of polyphenols was involved in the poor protein yield, we assayed the polyphenol content of *P. oceanica* dried leaves at the three depths (Fig. 1C). The samples from 2 and 15 m depths displayed a polyphenol concentration in the same range (6.75 ± 0.46 and 7.83 ± 0.18 mg Gallic Acid Equivalent (GAE)/mg chlorophyll, respectively) whilst the 26-m depth samples displayed a threefold lower polyphenol concentration (2.63 ± 0.07 GAE/mg chlorophyll). This result suggests that the polyphenol concentration could be a reason for the inefficient extraction of thylakoids or proteins from *P. oceanica*.

### Microscopy analysis of *P. oceanica* leaf tissues

**Fig. 2:**
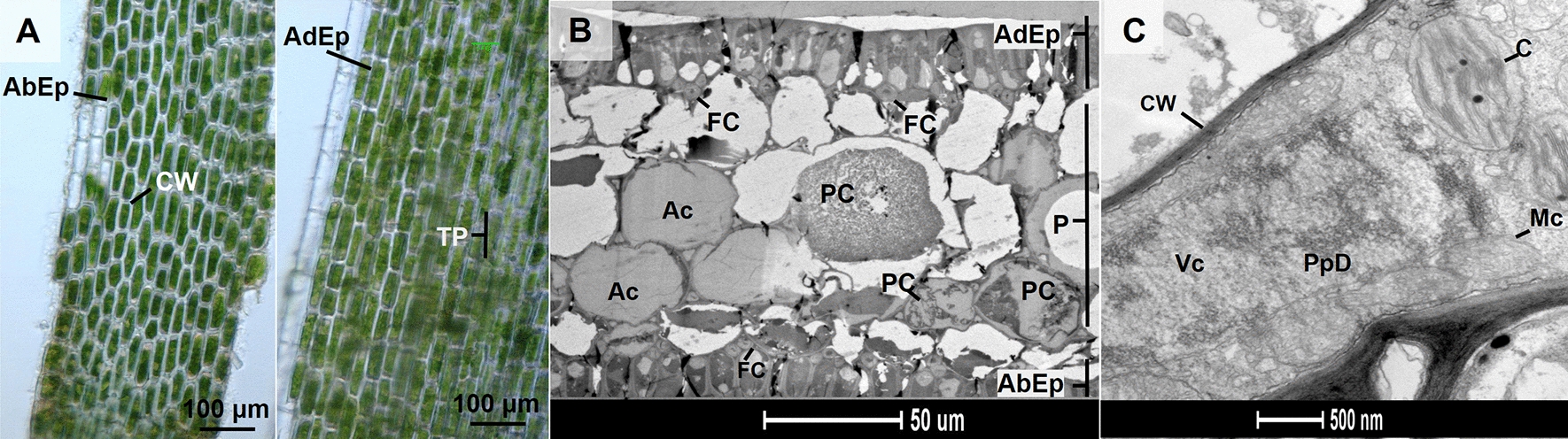
Organization and ultrastructure of leaf tissues in *P. oceanica*. **A** Low magnification microscopy observation of leaf tissue ultrastructure from *P. oceanica* (15 m depth). Left: view of the abaxial epidermis. Right: the abaxial epidermis was removed to expose underlying parenchyma. *AbEp* abaxial epidermis, *AdEp* adaxial epidermis, *CW* cell wall, *TP* transparent parenchyma. **B** Electron micrographs of a leaf transverse section (15 m depth). The lower (*AbEp*) and upper (*AdEp*) epidermis are separated by a parenchyma layer (aerenchyma). *Ac* aerenchyma, *FC* fusiform cells, *P* parenchyma, *PC* polyphenol cell. **C** Closed details of polyphenol cell (15 m depth) showing a polyphenol deposit in the vacuole. *CW* cell wall, *C* chloroplast, *Mc* mitochondria, *PpD* polyphenol deposits, *Vc* vacuole

In land plants, phenolic compounds are generally stored in vacuoles and cell walls. However, the polyphenols in *P. oceanica* are also confined in mesophyll-specialized structures [20, 21, 29], in contrast to those found in Australian Posidonia, which are deposited in the epidermis [30, 31]. Since optical microscopy did not provide a good visualization of the mesophyll structures (Fig. 2A), *P. oceanica* leaves (from 15 m depth) were observed using electronic microscopy (Fig. 2B). Observations revealed the presence of both low-contrasted aerenchyma cells (filled with gas) and cells characterized by a stronger grey coloration assigned to polyphenol deposit (Fig. 2B) [21]. The close-up view of a polyphenol-containing cell (PC) highlighted large vacuoles filled with polyphenols and numerous mitochondria suggesting high metabolic activity in polyphenol-containing cells (Fig. 2C)*.* These PCs could contribute to the formation of the cross-linking and gelation, observed during extraction of thylakoids.

### Effect of PEG and VitC on thylakoid membranes extraction from *P. oceanica*

**Fig. 3:**
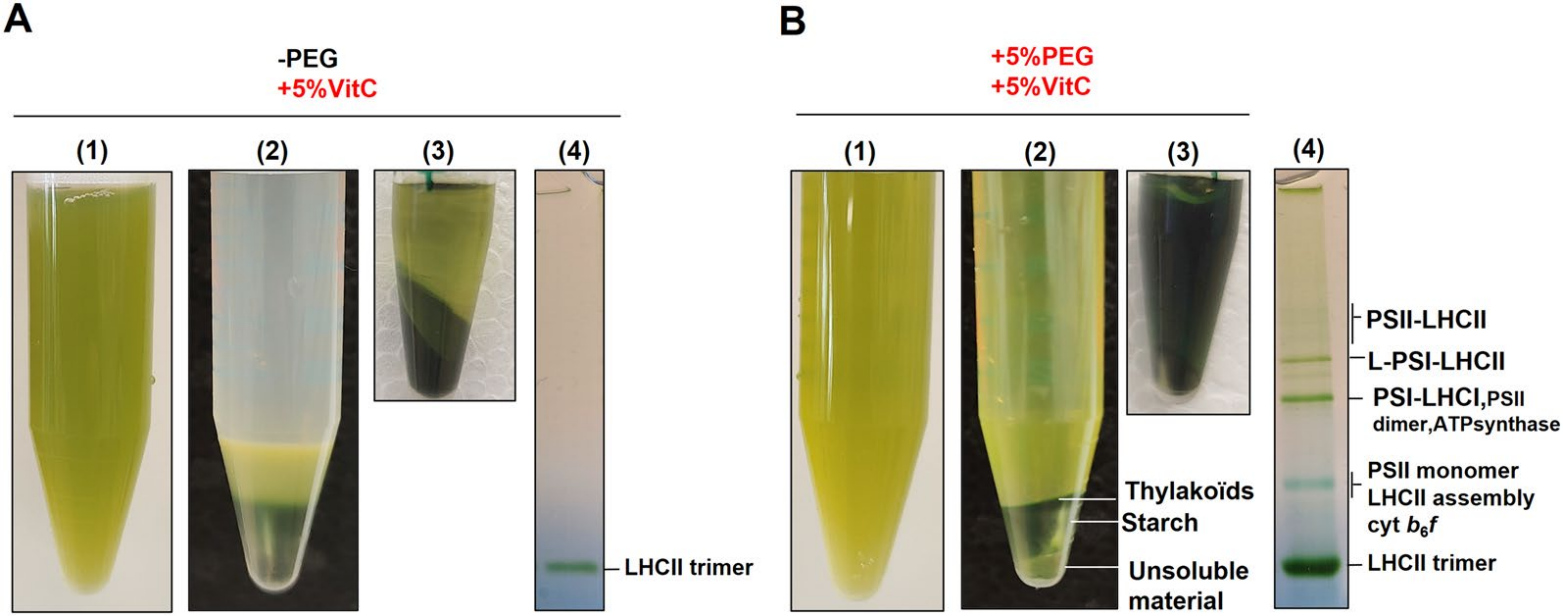
Effects of VitC and PEG on the quality of extracted thylakoids from *P. oceanica*. Leaves of *P. oceanica* (2 m depth) were ground in the presence of (5%) VitC in the absence (**A**) or presence (**B**) of (5%) PEG in the buffer. (1) Sample of the filtered ground leaf mixture. (2) Final pellet after resuspending the storage buffer. (3) Centrifuged pellet of the extracted thylakoids solubilized in 2% α-DDM per mg/mL of chlorophyll (20 mg DDM for 1 mg chlorophyll). (4) The protein content of the solubilizate analyzed by BN-PAGE

In an attempt to neutralize the effects of polyphenols, we extracted thylakoids with an extraction buffer supplemented with 5% VitC (concentration arbitrarily chosen for this assay) to prevent polyphenol oxidation and thus quinone formation. The ground leaf mixture appeared much clearer and greener (Fig. 3A left) than in the absence of VitC (Fig. 1A left). However, as shown in Fig. 3A (middle), the thylakoids were still trapped in mucilage and poorly solubilized (Fig. 3A right). To prevent reticulation of polyphenols and mucilage formation, we tested several conditions for extracting thylakoids such as NaCl instead of sorbitol, adding 5% milk powder or a reducing agent such as DTT, and acidic (pH 5) or alkaline (pH 8.5) buffers. None of these approaches were successful (data not shown). Furthermore, even the physical separation of thylakoids from mucilage using Percoll gradient centrifugation did not provide satisfactory results (data not shown). Therefore, we continued our assays focusing on the action of PEG together with VitC by adding 5% PEG4000 and 5% VitC in the grinding buffer (concentrations of chemicals were arbitrarily chosen for this assay). Using the PEG and VitC Protocol (PVC protocol), the pellet after centrifugation appeared differentiated into three layers, the dark green upper one, being the thylakoid membranes (Fig. 3B middle). After DDM treatment, the sample was successfully solubilized (Fig. 3B right), and photosynthetic complexes could be separated using BN-PAGE (compare with Figs. 1A and 3A right). We noted a similar yield across all species with the implementation of the PVC protocol (Additional file 2: Fig. S1).

### Effect of vitamin C and PEG on the extraction of total proteins from *P. oceanica* leaves

**Fig. 4:**
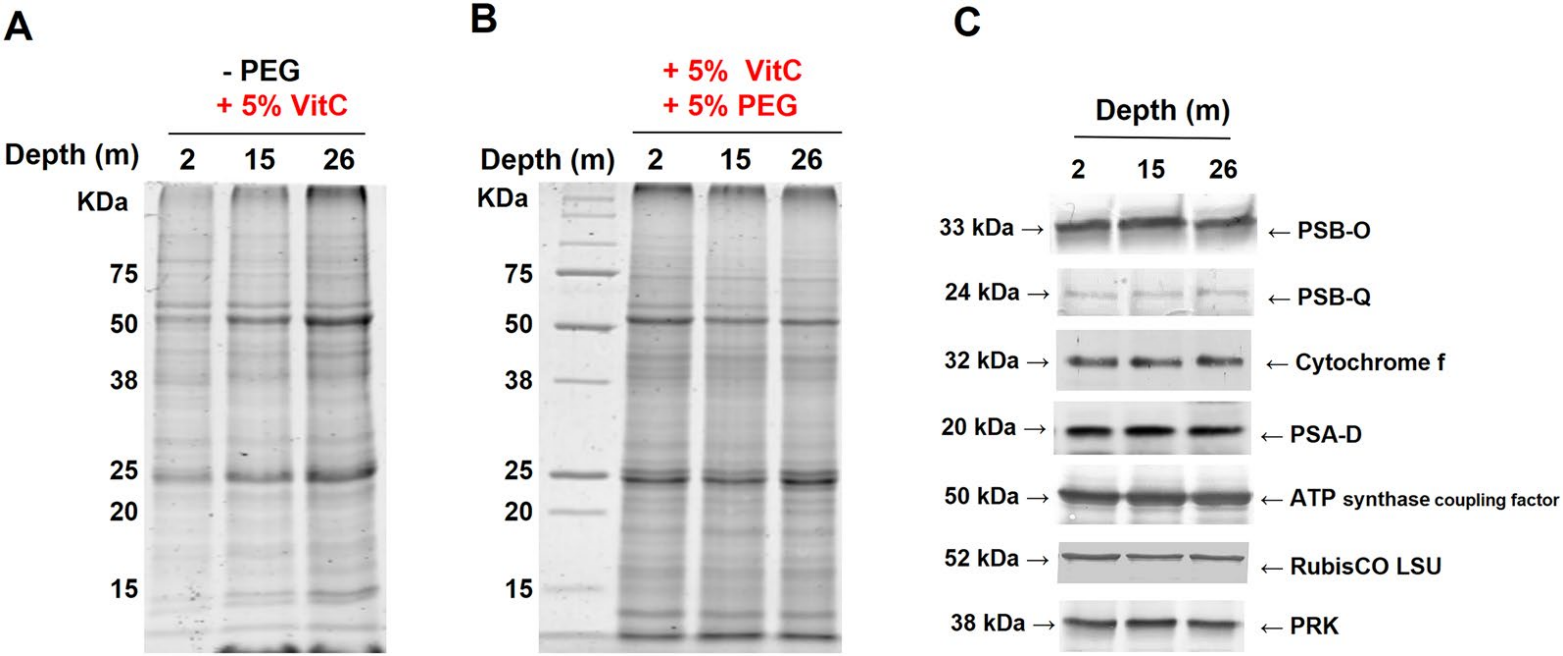
SDS-PAGE and immunoblot analyses of *Posidonia oceanica* leaf proteins from samples collected at different depths. Coomassie-blue-stained SDS-PAGE gels of total proteins extracted from *P. oceanica* leaves using laemmli denaturing buffers containing VitC (5%) without PEG (**A**) or with PEG (5%) (**B**). **C** Immunoblot analysis of photosynthetic proteins extracted from leaves in the presence of vitamin C (5%) and PEG (5%). Extraction was carried out using equal amounts of leaf material ground in denaturing buffer and equal volumes of protein extracts were loaded on the gels. For immunoblot analyses, equal protein amounts were loaded based on quantity evaluation from the scanning of Coomassie-blue stained gel

Total protein isolation from *P. oceanica* leaves was performed as shown in Fig. 1B but with the addition of VitC and/or PEG in the extraction buffer. Adding 5% VitC greatly improved the extraction procedure since proteins were successfully obtained from the samples harvested at different depths and bands could be observed in the gel (Fig. 4A). When extraction was performed in the presence of both 5% VitC and 5% PEG (Fig. 4B), very similar protein patterns in terms of content and band intensity were shown for the three samples**.** Furthermore, the resolution of the bands was slightly increased. Similarly, to that observed for thylakoid extraction (Fig. 3B), these results highlighted the beneficial effect of the presence of both VitC and PEG for preparing *P. oceanica* total protein extracts and their action on polyphenols irrespective of the extraction conditions (native or denaturing). We then aimed to validate this procedure by performing immunoblot analyses of protein extracts using various antibodies raised against photosynthetic proteins from land plant species involved in either the electron transfer chain or the Calvin-Benson cycle (Fig. 4C). Cross-reactions were observed for several of these sera. Proteins from PSII, PSI, cytochrome *b*_6_*f****,*** and ATP synthase complexes were probed in *P. oceanica* extracts. Similar abundances of these thylakoid proteins were noticed in the three types of samples. Similar amounts of RubisCO large subunit and phosphoribulokinase (PRK) were also revealed in these extracts. Altogether, these analyses validate the use of VitC and PEG for preparing *P. oceanica* protein extracts suitable for immunological experiments.

### Optimization of PEG and VitC concentrations and selection of the native gel system

**Fig. 5:**
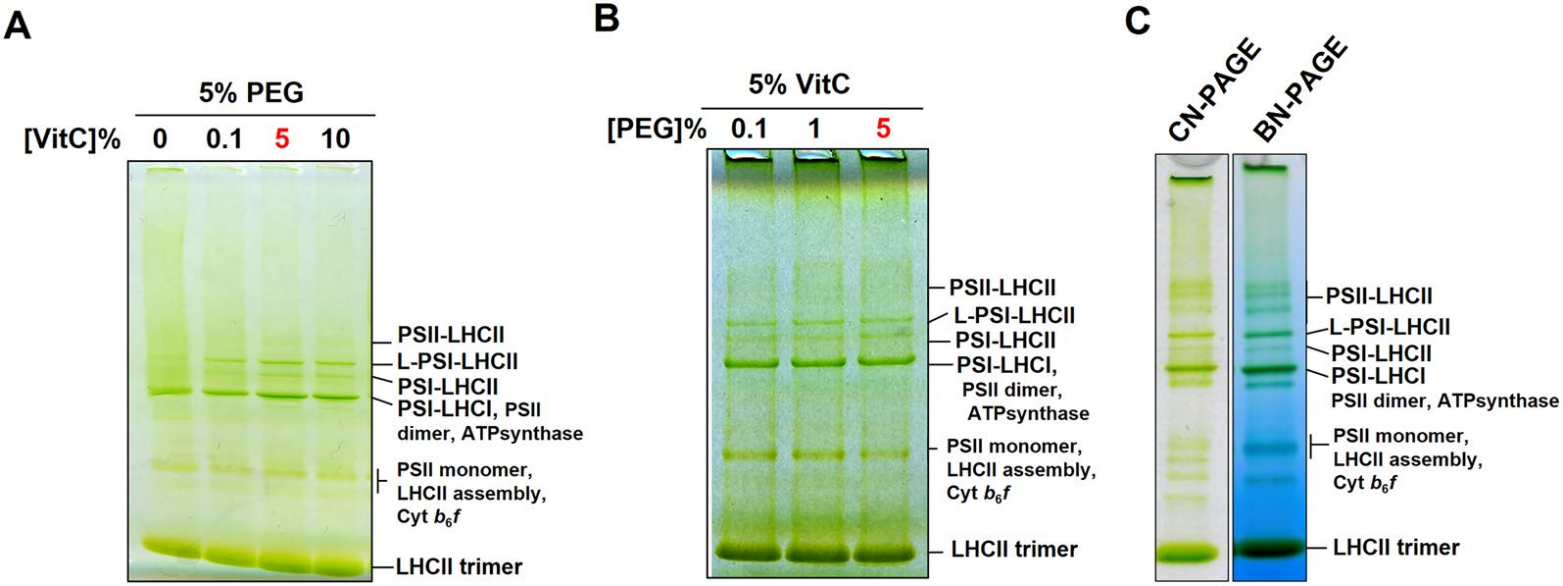
Determination of the optimal conditions for thylakoid extraction and separation of photosynthetic complexes from *P. oceanica*. **A** CN-PAGE of solubilized membrane from 26 m depth plants extracted with different concentrations of VitC (0–10%) in the presence of 5% PEG. The membranes were solubilized using a mixture of 1% β-DDM + 1% digitonin and 5 of μg chlorophyll was loaded onto the gel. **B** CN-PAGE of solubilized membrane extracted from 26 m depth plants with different concentrations of PEG (0.1–5%) in the presence of 5% VitC. The membranes were solubilized using a mixture of 1% β-DDM + 1% digitonin and 5 μg of chlorophyll was loaded onto the gel. **C** CN-PAGE vs BN-PAGE of 2% α-DDM solubilized membranes from 26 m depth plants. The thylakoid membranes were extracted using the optimized PVC protocol (5% PEG and 5% VitC)

While several photosynthetic complexes were extracted with the new protocol, the PSII-LHCII supercomplexes were weakly distinguishable in the BN-PAGE profile (Fig. 3B). To solve this problem, we tried to optimize the working concentration of PEG and VitC. For this purpose, *P. oceanica* thylakoid membranes were extracted using a gradually increasing concentration of VitC in the presence of 5% PEG. Better separation of solubilized complexes by CN-PAGE was found to correlate with increasing VitC concentration (Fig. 5A). In the absence of VitC, a smeared migration profile was noticed. When VitC concentration was raised to 0.1%, supercomplexes still appeared smeary but were better resolved than without VitC. The best band resolution was observed at 5% and 10% of VitC. While PSII-LHCII bands were still weak and smeary (suggesting poor solubilization of PSII), the well-separated bands highlighted the presence of PSI-LHCI, free LHCII trimeric antennae, and two PSI-(LHCI-)LHCII supercomplexes as determined by 2D Urea-PAGE analysis (Additional file 2: Fig. S2). Adding 0.1% PEG, in the presence of 5% VitC, solubilizes the thylakoids with supercomplexes being visible in CN-native gels (Fig. 5B). The best band resolution was obtained by raising concentration from 1% up to 5%. Finally, the concentrations used at the outset of the trials (5% of each compound) were used for the rest of the study. As proper concentrations of PEG and VitC were determined, the best native gel system was determined to separate and visualize the photosystem supercomplexes. When using 2% α-DDM, separating photosystem supercomplexes using a Blue-Native PAGE system allowed a better solubility, separation, and visibility of PSII-LHCII (Fig. 5C) in line with data from [1].

### Oxygen production in thylakoids extracted with the PVC protocol

We assessed the integrity of the OEC (Oxygen Evolving Complex) of PSII by measuring the oxygen production rate of thylakoids from *P. oceanica* (2 m), extracted with or without PEG and VitC. The results revealed that thylakoids extracted without PEG and VitC, or with VitC alone, exhibited a null oxygen production. However, employing the PVC protocol allowed us to measure a maximum oxygen production rate (OPR) of nearly 22.6 ± 5.7 µmol O_2_/mL/h/mg chlorophyll in *P. oceanica*, which is lower than the rate measured in *A*. *thaliana.* In the last species, a maximal rate of 81.9 ± 4.2 µmol O_2_/mL/h/mg chlorophyll was recorded in thylakoids extracted with the PVC protocol (Table 1, Additional file 2: Fig. S3). These findings indicate that the PVC protocol preserves OEC activity in *P. oceanica* and *A. thaliana*.

**Table 1:** Maximal oxygen production rate from *A. thaliana* thylakoids *(PVC protocol)* and *P. oceanica* (2 m) thylakoids

	µmol O_2_/h/mg chl
*P. oceanica* (− PEG − VitC)	0.0
*P. oceanica* (− PEG + 5%VitC)	0.0
*P. oceanica* (PVC protocol)	22.6 ± 5.7
*A. thaliana* (PVC protocol)	81.9 ± 4.2

Maximal OPR from *A. thaliana* and *P. oceanica* thylakoids extracted with the PVC protocol or with the conventional protocol supplemented with or without 5% VitC as indicated. The OPR was normalized by the quantity of chlorophyll (Additional file 2: Fig. S3). All Data are shown as the mean of three technical replicates ± SD

### Extraction of thylakoids from various plant species using the PVC protocol

Since the addition of PEG and VitC enabled the proper extraction of thylakoids in *P. oceanica*, the procedure was extended to other plant species known for their agronomic, economic, or ecological value, and which are different in terms of growth environment, morphology, and polyphenol content: *Quercus pubescens* (oak), *Vitis vinifera* (grapevine) and *Ocimum basilicum* (basil). The model plant *Arabidopsis thaliana* was also used as a control.

### BN-PAGE analysis of photosystem supercomplexes from various plant species using the PVC protocol

For each species, thylakoids were extracted using either the conventional or the PVC protocol.

**Fig. 6:**
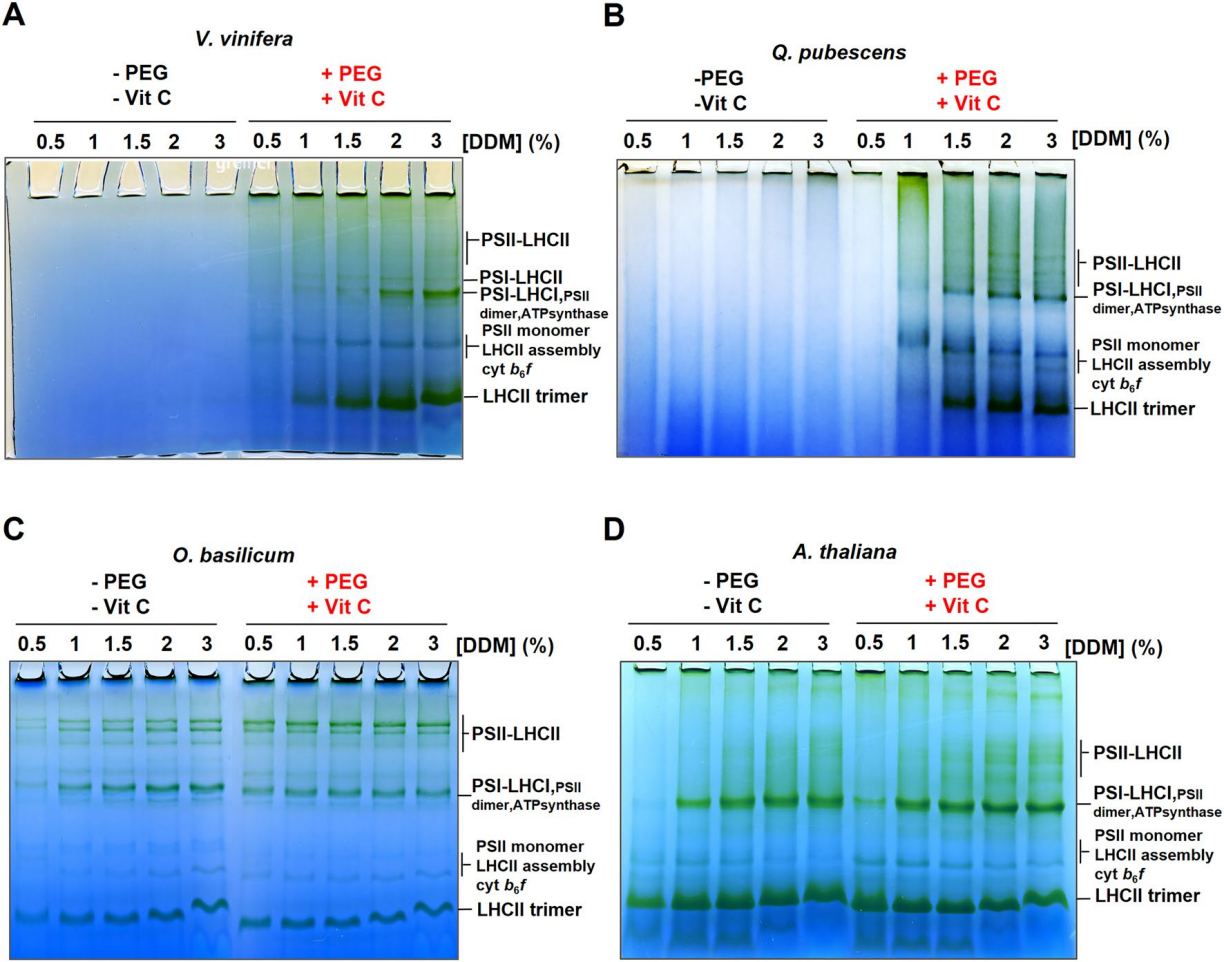
BN-PAGE of solubilized photosynthetic membranes from various leaf plant species extracted using the conventional and PVC protocols. BN-PAGE of solubilized thylakoids from **A** *V. vinifera*, **B** *Q. pubescens*, **C** *O. basilicum*, **D** *A. thaliana.* For each tested species, thylakoids were extracted with the conventional and the PVC protocol (5% PEG and 5% VitC in the grinding buffer). Thylakoids were solubilized with increasing α-DDM concentrations (0.5–3% per mg/mL of chlorophyll). The same volume of solubilized samples was loaded into the gel wells

As observed with *P. oceanica* (Fig. 1), *V. vinifera* and *Q. pubescens* supercomplexes were not or poorly solubilized when thylakoids were extracted without PEG and VitC even with a strong DDM concentration (3%) (Fig. 6A, B left). When using the PVC protocol, thylakoids were partially solubilized in the presence of 1% DDM as shown by smeared bands in the gels. By increasing the concentration of DDM to 2%, the complexes were visible as well-resolved distinct bands (Fig. 6A, B right). At first glance, the migration pattern was somewhat different between *V. vinifera* and *Q. pubescens*, but 2D-PAGE analysis (Additional file 2: Fig. S4) confirmed the presence of free LHCII, PSI-LHCI, PSII-LHCII supercomplexes and of a band assigned to the PSI-LHCI-LHCII complex in *V. vinifera*. While free LHCII was observed in the gel using the conventional protocol with *V. vinifera* extracts, no complex was observed in the *Q. pubescens* migration profile when using the same protocol. On the other hand, the PVC protocol allowed the solubilization of all photosynthetic complexes in the thylakoids, demonstrating its efficiency. In *A. thaliana,* the migration profile was similar when using conventional or PVC protocol, but differed in band resolution (Fig. 6D). Indeed, while the PSII supercomplex bands appeared to spread out using 3% DDM with the conventional protocol, these bands were already resolved from 2% DDM when using the PVC protocol. In *O. basilicum*, few differences were observable between the two procedures despite that the PSI-LHCI bands and free LHCII antennae appeared greener with the conventional protocol (Fig. 6C) whereas, with the PVC protocol, the highest PSII-LHCII bands appeared more intense (Additional file 2: Fig. S4). This might suggest better grana solubilization when using the PVC method or better stability of supercomplexes during solubilization. However, the slight differences observed, between protocols in the resolution of PSII-LHCII bands for *O. basilicum* and *A. thaliana* might be related to our working conditions, i.e. the use of frozen leaves. Many reports show nice native gels from fresh tissue thylakoids are available for *A. thaliana* [1], but using the PVC protocol is not necessary for this species.

### Soluble polyphenol content in leaves from various plant species

**Fig. 7:**
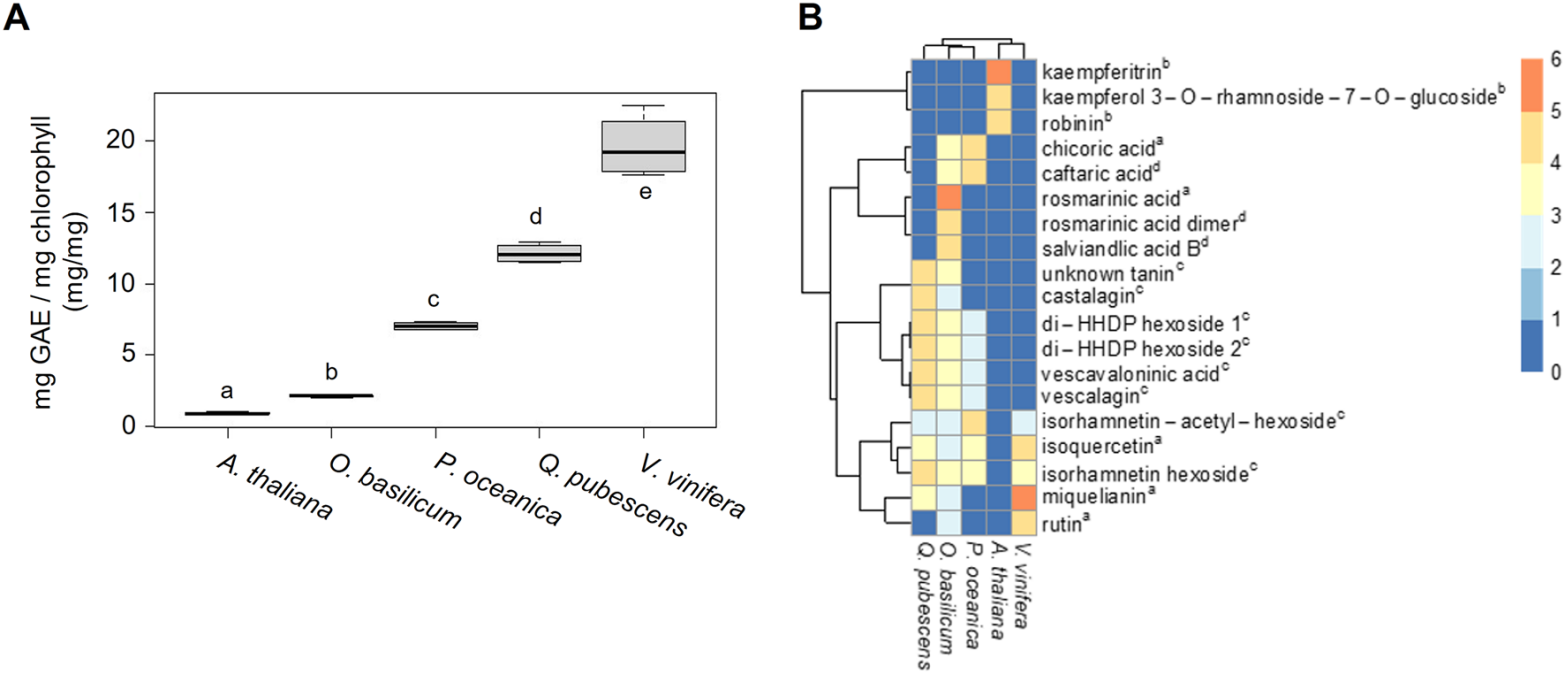
Soluble polyphenol content in leaves from selected plant species. **A** The measured polyphenol content of the extract was normalized by the total chlorophyll content of freeze-dried leaf material. Different letters indicate significant differences at *P* < 0.05, as determined using the Shapiro–Wilk normality test followed by the student’s t-distribution analysis. Data are represented as the median, quartiles, and border values of 4 technical replicates. Data and calculations are presented in Additional file 1: Data S1. **B** Phenolic compounds profiling. Heatmap of the major phenolic compounds (identified with available standards (a) or annotated from literature (b: [45], c: [47], d: [48]) in the five species (*Q. pubescens*, *O. basilicum*, *P. oceanica*, *A. thaliana*, *V. vinifera*). Common logarithm of the peak area means (area/mL) for each condition (five technical replicates) and each metabolite is expressed per one microliter of the extract solution injected (one mg of the starting plant dry material per mL). Colors in the heatmap are related to the sequential logarithmic scale presented in the right panel (0: not detected)

We hypothesized that polyphenols would have inhibitory effects on thylakoid and protein extraction independent of their localization in parenchyma cells (*P. oceanica*) or epidermis cells (land plants). To test this hypothesis, the soluble polyphenol content of dried leaves from *P. oceanica* (15 m depth) *Q. pubescens*, *V. vinifera*, *O. basilicum* and *A. thaliana* was determined (Fig. 7A). As these plant species do not share the same leaf structure (e.g., leaf thickness, the structure of vascular tissues, epidermis, and parenchyma organization), the polyphenol content is likely to differ (Fig. 2A and Additional file 2: Fig. S5). The soluble polyphenol content was therefore related to the leaf chlorophyll content (Fig. 7 and Additional file 2: Fig. S6). Of the five species tested, *V. vinifera* had the highest polyphenol content (19.6 ± 2 mg GAE/mg Chl), followed by *Q. pubescens* (12.1 ± 0.7 mg GAE/mg Chl) and *P. oceanica* (7.0 ± 0.3 mg GAE/mg Chl). In contrast, *O. basilicum* (2.1 ± 0.1 mg GAE/mg Chl) and *A. thaliana* (0.9 ± 0.1 mg GAE/mg Chl) displayed the lowest contents. *Q. pubescens* and *V. vinifera*, which exhibit the highest polyphenol contents, are the species, for which the extraction of thylakoids required the PVC protocol like *P. oceanica*. Given that the inhibitory effects of polyphenols may be associated with specific compounds, the polyphenol composition of the five plants was determined. Figure 7B shows that *O. basilicum* leaves contain a wide array of polyphenolic compounds compared to the other species (16 of the 19 tested molecules). However, the PVC protocol was not required for thylakoid extraction from this plant. Moreover, all polyphenols identified in *P. oceanica* (9 polyphenols), *V. vinifera* (5), and *Q. pubescence* (10) were detected in *O. basilicum*. This result suggests that the concentration of polyphenols, rather than their specific chemical composition, is a critical factor in preventing thylakoid membrane extraction.

## Discussion

In this study, we developed a protocol for the extraction of proteins and thylakoids from polyphenol-rich plants, for which conventional procedures are not suitable. Initially, as we suspected cross-reaction of cell wall components with thylakoids, we performed pretreatments of leaves using enzymes acting directly against the cell wall polymers (e.g., lignin and cellulose, data not shown). These treatments did not improve the extraction of thylakoids and the quality of the final protein preparation. Several reports showed that polyphenols are characterized by gelling properties (reticulation and cross-linking) and high sensitivity to oxidation [14, 16–18]. To neutralize the oxidation of polyphenols leading to the formation of highly oxidizing quinones, the conventional extraction protocols for thylakoids and leaf proteins were first modified by adding VitC (5%), which is a soluble and inexpensive antioxidant. The optimal VitC concentration probably depends on other factors such as the polyphenol content and properties, which then determine the ability of oxidized polyphenols and VitC to establish a redox equilibrium. For example, in other marine plant species tested, the VitC concentration needs to be raised to 10%, (data not shown). The redox equilibrium reached through the optimal VitC concentration may favor the redox reaction of quinones with VitC molecules instead of proteins and other leaf cell components [32–34]. However, VitC alone was not sufficient to obtain proper thylakoid preparations as we also encountered issues attributed to the reticulation of polyphenols. Indeed, the cross-linking reaction of polyphenols leads to the formation of a mucilaginous mass trapping the thylakoids (Fig. 1). We were able to inhibit this deleterious process by adding 0.1 up to 5% PEG to the grinding buffer (5% being the concentration we use for our work in *Posidonia oceanica*). PEG molecules own repeated ether groups all along the molecules, which bind to the hydroxyl groups of phenolic compounds via hydrogen bonds, thereby blocking their very high reactivity [35, 36].

Moreover, in the plants studied in the present work, we have also shown that the concentration of polyphenols in leaves is an essential parameter for the successful preparation of the thylakoid membranes since we excluded the implication of specific polyphenol species (Fig. 7). This hypothesis is corroborated by the fact that polyphenol concentrations in the range of 1 to 2 GAE/mg chlorophyll were measured in *A. thaliana* and *O. basilicum*, species for which thylakoid extraction does not require the PVC protocol, while much higher levels were determined in *Posidonia*, oak, and grapevine. Interestingly, denaturing extraction of proteins in *Posidonia* in the absence of VitC and PEG led to very poor yield in samples collected at 2 and 15 m depth, which are characterized by polyphenol contents of ~ 7 GAE/mg chlorophyll (Fig. 1B). Extraction was much more efficient in the samples collected at 26 m depth, which display a lower polyphenol content of 2.6 GAE/mg chlorophyll. Those data suggest a threshold value in the range of this concentration, above which extraction of native or denatured proteins becomes difficult in *Posidonia.*

Other biological and metabolic barriers could also prevent the successful extraction of thylakoids in plant species characterized by very thick and leathery leaves such as conifers, palm trees, or olive trees (*Olea europaea*) [37], which exhibit an elevated wax content in the cuticle. Nevertheless, the optimized protocol works well on the various plant species tested in this study, particularly *Posidonia oceanica*, but also *Vitis vinifera* and *Quercus pubescens*, which are very rich in polyphenols. Moreover, the PVC protocol did not seem to decrease the extraction yield of thylakoids since we obtained a similar amount of chlorophyll per fresh weight using the two protocols in *O. basilicum* and *A. thaliana* (Additional file 2: Fig. S1).

Although we did not assess the efficacy of Vitamin C (Vit C) alone in the two additional species necessitating the PVC protocol (*Vitis vinifera* and *Quercus pubescens*), its application is unlikely to facilitate thylakoid extraction. VitC, being a small molecule unlike PEG, a substantial molecular weight polymer, prevents polyphenol cross-linking by capturing them through hydrogen bonds and van der Waals forces [16–18]. As demonstrated in Posidonia leaves (Fig. 3A), VitC alone fails to impede polyphenol cross-linking, rendering it ineffective for thylakoid isolation in Posidonia. Moreover, polyphenol levels are significantly higher in *Vitis vinifera* and *Quercus pubescens* compared to *P. oceanic*a (Fig. 7), suggesting that the use of VitC would not prevent polyphenol reticulation due to the elevated polyphenol concentrations in *Vitis vinifera* and *Quercus pubescens*.

## Conclusion

This protocol can be applied to a large number of species that stand out by a high polyphenol content since most plants synthesize polyphenols, especially under environmental constraints. Thus, such a protocol will allow improving the knowledge not only in the photosynthesis field but also in other areas of plant physiology and metabolism that could not be easily studied up to now in plants grown in natural conditions.

## Methods

### Samples collection

*Posidonia oceanica* individuals (sheat-bundles and leaves) were harvested in the south of Frioul Island, Marseille, France (Coordinate 43° 16′ 11″ N 5° 17′ 32″ E). The collections were performed in January 2019 and January 2021 between 9 and 11 a.m. at 2, 15, and 26-m depths. The samplings were carried out within the framework of a protected species exemption with a prefectural decree authorizing them*.*

Immediately after harvesting, plants were kept in the dark in cold seawater. Leaves were separated from bundles at 4 °C under green light, and epiphyte organisms were removed by washing with seawater. *A. thaliana* leaves were harvested from 4-week-old plants grown under 120 μmol photons/m^2^/s white light; *O. basilicum* stalks were purchased in a garden center store. *Q. pubescens* and *V. vinifera* leaves were harvested from a set of four different trees located in Luminy campus park (Marseille, France) and a vineyard (Aix-en-Provence, France, respectively. Young and adult-developing leaves were selected. Due to logistic constraints in sample collection, leaves from all species were frozen in liquid nitrogen following collection and stored at − 80 °C for further studies.

### Thylakoid membrane extraction

The thylakoid membrane extraction protocols further called “the conventional protocol” and “PVC protocol” (the PEG + vitamin C) were inspired by [38] with some modifications in the buffer composition. For optimization experiments using *Posidonia oceanica*, we used leaves from different growing depths. Detailed instructions about the complete extraction procedure are shown in Additional file 3: Method S1. The composition of the buffers and the main step of the procedure are described below:

- Thylakoid membranes were extracted by grinding leaves in two distinct buffers (GB). For the conventional protocol, the buffer was composed of 20 mM Tricine pH 7.8, 0.3 M sorbitol, 10 mM EDTA, 10 mM NaHCO_3_, 0.15% bovine serum albumin (BSA), 10 mM of phosphatase inhibitor NaF, 5 mM benzamidine, 5 mM caproic acid. For the PVC protocol, the grinding buffer described above was supplemented with 5% Polyethylene glycol 4000 (PEG4000) and 5% ascorbic acid (vitamin C, Sigma).
- Chloroplasts were then burst using a hypotonic buffer (25 mM Hepes–KOH pH 7.5, 25 mM sorbitol, 5 mM NaCl, 5 mM MgCl_2_, 5 mM KCl, 10 mM NaF, and 5 mM Benzamidine, 5 mM caproic acid, cOmplete™ Protease Inhibitor Cocktail).
- The extracted thylakoid membranes were stored in the storage buffer (50 mM Hepes pH 7.5, 0.3 M sorbitol, 10 mM NaCl, 5 mM MgCl_2_, 10 mM NaF, cOmplete™ Protease Inhibitor Cocktail).

### Isolation and separation of photosystem supercomplexes

#### Large pore native-PAGE preparation

Gels were prepared using a 40T and 3C acrylamide/bis-acrylamide (w/v) mixture. This represents 38.8 g of acrylamide added to 1.2 g of bis-acrylamide diluted in 100 mL distilled water. This T/C ratio was chosen according to [1]. Gels containing 3–12% (w/v) T, and 3% C were cast according to [1] with minor modification as no stacking gel was cast above the resolving gel (3–12%).

#### Thylakoid membrane solubilization

Thylakoid membranes were washed twice in a solubilization buffer (50 mM Bis–Tris pH 7, 20% glycerol, 20 mM EDTA, 10 mM NaF). The membranes were then diluted to 1 mg/mL of chlorophyll. Then, an equal volume of 1–6% (w/v) *n*-dodecyl-ɑ-d-maltopyranoside (DDM, Anatrace, Ref: D310HA), or detergents specified otherwise, prepared in the solubilization buffer was added. Membranes and detergents were then incubated at 4 °C for 20 min under gentle agitation. After solubilization, samples were centrifuged for 30 min at 18,000×*g* to remove insoluble materials. The supernatant was then used for Clear-Native and Blue Native-PAGE sample preparation.

#### Native PAGE sample preparation

Large pore Clear Native-PAGE (referred to as CN-PAGE) and Large pore Blue Native-PAGE (referred to as BN-PAGE) were carried out similarly in [1] and the solubilizate was supplemented with 1/10 volume of Serva Blue loading buffer for BN-PAGE (100 mM Bis–Tris methane pH 7.0, 0.5 M amino-n-caproic acid, 30% (w/v) sucrose and 50 mg/mL Serva Blue G) or 1/10 DOC (Deoxycholate) loading buffer for CN-PAGE (100 mM Bis–Tris methane pH 7.0, 0.5 M amino-*n*-caproic acid, 30% (w/v) sucrose and 5% DOC).

#### Separation of photosystem supercomplexes by lp-Clear and Blue native-PAGE

An anode buffer (25 mM BisTris, 25 mM Tricine, pH 7.0) was used for electrophoresis for both lp-CN and lp-BN gels. For lp-CN, the cathode buffer was composed of 50 mM BisTris-HCl, 50 mM Tricine, pH 7.0, and supplemented with 0.015% DDM and 0.05% DOC (Deoxycholate). For lp-BN, the cathode buffer was composed of 50 mM BisTris-HCl, 50 mM Tricine, pH 7.0, and supplemented with 0.015% DDM and 0.01% Serva Blue G 250. About 5–10 μg of chlorophyll were loaded onto the gel wells. Electrophoresis was performed at 4 °C for 7 h. The current intensity was fixed and adjusted so that at the start of the run, the voltage was around 70 V.

#### Second dimension urea-PAGE separation of supercomplexes isolated by BN-PAGE

The lanes of interest were cut out from 1st Dimension CN or BN-PAGE gels and incubated for 10 min in Laemmli denaturing sample buffer (125 mM Tris–HCl, pH 6.8, 20% glycerol, 2% SDS, 100 mM DTT, 0.01% bromophenol blue). The lanes were further loaded on the top of a 13% acrylamide/bis-acrylamide (37.5/1) resolving gel and fixed with agarose (0.125 M Tris–HCl, pH 6.8, 2% agarose, 0.001% bromophenol blue). For the run, the anode and cathode chambers were filled up with Laemmli running buffers (25 mM Tris–HCl, pH 8.3, 0.192 M glycine, 0.1% SDS). The migration was performed for approximately 2 h at 90 V.

#### Leaf total protein extraction

*Posidonia oceanica* leaves were ground in liquid nitrogen and the powder (*ca*. 1 g) was resuspended in 2 ml of a buffer containing 50 mM Tris–HCl pH 8, 1% SDS, 1 mM PMSF, 50 mM β-mercaptoethanol and supplemented or not with 5% (w/w) vitamin C (Sigma) and/or 5% PEG-4000 (Sigma) to prepare extracts containing soluble and membrane proteins. After homogenization for a few minutes and vigorous shaking for at least 3 h at room temperature, extracts were centrifuged at room temperature for 30 min at 18,000×*g*. Four volumes of acetone were added to the supernatant to precipitate proteins at − 20 °C overnight. After centrifugation, the protein pellet was then let to dry and resuspended in the Laemmli denaturing buffer.

#### SDS-PAGE protein electrophoresis and Western blot analysis

Proteins were separated in 13% acrylamide SDS-PAGE gel either for staining using Coomassie Blue (Imperial™ Protein Stain, Thermo Scientific) or for electroblotting onto 0.45 µm nitrocellulose (Pall Corporation) to perform immunoblot analysis. To evaluate protein yield in equal extract volumes, SDS-PAGE gels were carried out to quantify band intensity, following staining with Imperial Coomassie Blue (ThermoFisher), using the ‘Odyssey Infrared Imager’ scanning system at 680 nm (Licor, Lincoln, NE, USA). Membranes were stained using Ponceau Red (Sigma) to ensure homogenous transfer and loading of lanes (Additional file 4). Antibodies against ribulose-1,5-bisphosphate carboxylase/oxygenase (RubisCO) and ATP synthase coupling factors (kindly provided by Dr. GH Schmid, University of Bielefeld, Germany) were diluted 1:10,000, and those raised against cytochrome f, PsaD and PRK (kindly provided by Dr. X. Johnson, BIAM, Aix-Marseille Université, France) were used diluted 1:5000, 1:5000 and 1:2000, respectively. The sera against PsbO and PsbQ (PSII subunits) were purchased from Agrisera (Vännäs, Sweden) and used diluted 1:5000. Bound antibodies were detected using either an anti-rabbit immunoglobulin G coupled to alkaline phosphatase (Sigma) or a goat anti-rabbit secondary antibody coupled to a fluorescent molecule at a dilution of 1:10,000 (Alexa Fluor 680, Invitrogen) using the ‘Odyssey Infrared Imager’ at 680 nm (Licor, Lincoln, NE, USA).

### Leaf chlorophyll extraction and assay

Leaves were ground in liquid nitrogen using a mortar and pestle, and the powder was then freeze-dried. The powder was further pulverized using a Vibro-crusher. The chlorophyll was then extracted in 80% acetone (buffered with 5 mM Hepes pH 7.0) containing 0.5% ascorbic acid (VitC) to avoid polyphenol oxidation. The acetone (80%) + VitC buffer was prepared freshly to avoid VitC oxidation. The chlorophyll concentration of the purified thylakoids and the leaf extracts were measured as described in [39, 40].

### Extraction and content determination of leaf hydrosoluble polyphenols

Polyphenols were extracted from the same powder used for chlorophyll extraction as described in [41]. The powder (50 or 100 mg) was mixed in a 15 mL tube containing 2.4 mL of 50% ethanol in water. The 15 mL tube was filled with water and placed in a microwave. The sample was then heated at 100 W power for 5 min. Every 30 s, the sample was homogenized and put back into the microwave for another 30-s run. Quantification of the extracted polyphenols was performed as described in [42]. The standard curve was performed using gallic acid. The quantity of extracted polyphenols was expressed in terms of Gallic Acid Equivalent (GAE) per dry leaf weight or normalized to the total chlorophyll content per dry weight.

### Polyphenol profiling

Extraction and UHPLC-ESI-HRMS profiling analysis was carried out according to [46]. The major phenolic compounds in each species were identified with available standards or annotated with bibliographic data [45–48]. Common logarithm of the peak area means (> 19,000 area/mL) for each condition (five technical replicates) and for each metabolite expressed per one microliter injected of the extract solution (one mg of the starting plant dry material per mL). Heatmaps were performed with the R software (version 3.6.3, company Foundation for Statistical Computing, Vienna, Austria) using the heatmap package (version 1.0.12). Heatmap data was clustered using Ward’s method.

### Electron microscopy

Samples were prepared as described in [43]. Grids were observed under the Tecnai G2 electron microscope.

### Clark-type O_2_ measurement on isolated thylakoids

Light-induced O_2_ evolution was measured using a Clark-type electrode (Hansatech Instruments, England; Oxytrace + software) under 1500 µmol photons m^−2^ s^−1^ of white LED illumination. Calibration followed Oxytrace + recommendations: initially, the maximal oxygen concentration in an O_2_-saturated solution (storage buffer) was determined, followed by degassing with N_2_ bubbling until a minimal electrode potential indicated zero O_2_ concentration. Thylakoids, diluted to 10 μg/ml chlorophyll in storage buffer, were introduced into the measurement chamber in a volume of 2 ml. For *P. oceanica* thylakoids, 50 mM HCO_3_^−^ was added. Electron acceptors DCBQ (Dichlorobenzoquinone) and FeCy (Potassium ferricyanide) were incorporated at final concentrations of 250 µM and 500 µM, respectively, with NH_4_Cl at 4 mM to ensure membrane H^+^ proton permeability. The reaction medium was thoroughly degassed with nitrogen until the oxygen concentration reached zero, followed by hermetic sealing of the chamber. Degassing completion preceded the start of recording and illumination activation.

### Supplementary Information

**Additional file 1.** Data set 1: Leaf chlorophyll and polyphenols content determination. Summary table data set 1: Summary of computed and normalized data from data set 1. Data Set 2 : Determination of the yield of extracted thylakoids chlorophyll.

**Additional file 2: Figure S1.** Chlorophyll extraction yield using conventional and PVC protocols from selected plant species. **Figure S2.** 2D Urea-PAGE for the identification of *P. oceanica* supercomplexes isolated by CN-PAGE. **Figure S3.** Oxygen production rate from *A. thaliana* thylakoids (PVC protocol) and *P. oceanica* (2 m) thylakoids (conventional protocol, conventional protocol + 5% VitC and PVC protocol). **Figure S4.** 2D Urea-PAGE for the identification of peptides of supercomplexes isolated by BN-PAGE from various plant species. **Figure S5.** Organization and ultrastructure of leaf tissues from *A. thaliana* and *Q. pubescens*. **Figure S6.** Leaf chlorophyll content from various plant species.

**Additional file 3: Method S1.** Detailed protocol for the extraction of the thylakoid membranes from *P. oceanica* and various plant species.

**Additional file 4.**

 Original and uncropped gels, blots and pictures.

## Data Availability

Data generated or analyzed during this study are included in this published article or are available upon request.

## References

[1] Järvi S, Suorsa M, Paakkarinen V, Aro E-M (2011). Optimized native gel systems for separation of thylakoid protein complexes: novel super- and mega-complexes. Biochem J.

[2] Fristedt R, Willig A, Granath P, Crèvecoeur M, Rochaix J-D, Vener AV (2010). Phosphorylation of photosystem II controls functional macroscopic folding of photosynthetic membranes in *Arabidopsis*. Plant Cell.

[3] Caffarri S, Kouřil R, Kereïche S, Boekema EJ, Croce R (2009). Functional architecture of higher plant photosystem II supercomplexes. EMBO J.

[4] Fei J, Wang Y-S, Cheng H, Su Y-B (2021). An efficient protein extraction method applied to mangrove plant *Kandelia obovata* leaves for proteomic analysis. Plant Methods.

[5] D’Esposito D, Orrù L, Dattolo E, Bernardo L, Lamontanara A, Orsini L, Serra IA, Mazzuca S, Procaccini G (2016). Transcriptome characterisation and simple sequence repeat marker discovery in the seagrass *Posidonia oceanica*. Sci Data.

[6] Dattolo E, Gu J, Bayer P, Mazzuca S, Serra IA, Spadafora A, Bernardo L, Natali L, Cavallini A, Procaccini G (2013). Acclimation to different depths by the marine angiosperm *Posidonia oceanica*: transcriptomic and proteomic profiles. Front Plant Sci.

[7] Méchin V, Damerval C, Zivy M, Thiellement H, Zivy M, Damerval C, Méchin V (2007). Total protein extraction with TCA-acetone. Plant proteomics: methods and protocols.

[8] Jiang Y, Duan X, Qu H, Zheng S, Caballero B, Finglas PM, Toldrá F (2016). Browning: enzymatic browning. Encyclopedia of food and health.

[9] Mesquita VLV, Queiroz C, Eskin NAM, Shahidi F (2013). Chapter 10—Enzymatic browning. Biochemistry of foods.

[10] Chapter 17—Redox phenomena in must and wine. In: Moreno J, Peinado R, editors. Enological chemistry. San Diego: Academic Press; 2012. p. 289–302. ISBN 978-0-12-388438-1.

[11] Teoh ES (2015). Secondary metabolites of plants. Medicinal orchids of Asia.

[12] Šamec D, Karalija E, Šola I, Vujčić Bok V, Salopek-Sondi B (2021). The role of polyphenols in abiotic stress response: the influence of molecular structure. Plants.

[13] Tuladhar P, Sasidharan S, Saudagar P, Jogaiah S (2021). 17—Role of phenols and polyphenols in plant defense response to biotic and abiotic stresses. Biocontrol agents and secondary metabolites.

[14] Quideau S, Deffieux D, Douat-Casassus C, Pouységu L (2011). Plant polyphenols: chemical properties, biological activities, and synthesis. Angew Chem Int Ed.

[15] Sang S, Hou Z, Lambert JD, Yang CS (2005). Redox properties of tea polyphenols and related biological activities. Antioxid Redox Signal.

[16] Chen C, Li D, Yano H, Abe K (2019). Bioinspired hydrogels: quinone crosslinking reaction for chitin nanofibers with enhanced mechanical strength via surface deacetylation. Carbohydr Polym.

[17] Jimtaisong A, Saewan N (2018). Plant-derived polyphenols as potential cross-linking agents for methylcellulose-chitosan biocomposites. Solid State Phenom.

[18] Zhao Y, Sun Z (2017). Effects of gelatin-polyphenol and gelatin-genipin cross-linking on the structure of gelatin hydrogels. Int J Food Prop.

[19] Morris H, Brodersen C, Schwarze FWMR, Jansen S (2016). The parenchyma of secondary xylem and its critical role in tree defense against fungal decay in relation to the CODIT model. Front Plant Sci.

[20] Dumay O, Costa J, Desjobert J-M, Pergent G (2004). Variations in the concentration of phenolic compounds in the seagrass *Posidonia oceanica* under conditions of competition. Phytochemistry.

[21] Colombo PM, Rascio N, Cinelli F (1983). *Posidonia oceanica* (L.) Delile: a structural study of the photosynthetic apparatus. Mar Ecol.

[22] Grebe S, Trotta A, Bajwa AA, Suorsa M, Gollan PJ, Jansson S, Tikkanen M, Aro E-M (2019). The unique photosynthetic apparatus of pinaceae: analysis of photosynthetic complexes in *Picea abies*. J Exp Bot.

[23] Šuković D, Knežević B, Gašić U, Sredojević M, Ćirić I, Todić S, Mutić J, Tešić Ž (2020). Phenolic profiles of leaves, grapes, and wine of grapevine variety Vranac (*Vitis vinifera* L.) from Montenegro. Foods.

[24] Pandey A, Belwal T, Tamta S, Rawal RS (2021). Optimized extraction of polyphenolic antioxidants from the leaves of Himalayan oak species. PLoS ONE.

[25] Laugier E, Tarrago L, Vieira Dos Santos C, Eymery F, Havaux M, Rey P (2010). *Arabidopsis thaliana* plastidial methionine sulfoxide reductases B, MSRBs, account for most leaf peptide MSR activity and are essential for growth under environmental constraints through a role in the preservation of photosystem antennae. Plant J Cell Mol Biol.

[26] Gillet B, Beyly A, Peltier G, Rey P (1998). Molecular characterization of CDSP 34, a chloroplastic protein induced by water deficit in *Solanum tuberosum* L. plants, and regulation of CDSP 34 expression by ABA and high illumination. Plant J Cell Mol Biol.

[27] M’rah S, Ouerghi Z, Eymery F, Rey P, Hajji M, Grignon C, Lachaâl M (2007). Efficiency of biochemical protection against toxic effects of accumulated salt differentiates *Thellungiella halophila* from *Arabidopsis Thaliana*. J Plant Physiol.

[28] Bouchenak F, Henri P, Benrebiha F-Z, Rey P (2012). Differential responses to salinity of two *Atriplex halimus* populations in relation to organic solutes and antioxidant systems involving thiol reductases. J Plant Physiol.

[29] Messina CM, Arena R, Manuguerra S, Pericot Y, Curcuraci E, Kerninon F, Renda G, Hellio C, Santulli A (2021). Antioxidant bioactivity of extracts from beach cast leaves of *Posidonia oceanica* (L.) Delile. Mar Drugs.

[30] Kuo J (1978). Morphology, anatomy and histochemistry of the Australian seagrasses of the genus *Posidonia könig* (Posidoniaceae). I. Leaf blade and leaf sheath of *Posidonia australis* Hook F. Aquat Bot.

[31] Kuo J, den Hartog C, Larkum AWD, Orth RJ, Duarte CM (2006). Seagrass morphology, anatomy, and ultrastructure. Seagrasses: biology, ecologyand conservation.

[32] Sapper H, Kang SO, Paul HH, Lohmann W (1982). The reversibility of the vitamin C redox system: electrochemical reasons and biological aspects. Z Naturforsch.

[33] Kolniak-Ostek J, Oszmiański J, Wojdyło A (2013). Effect of l-ascorbic acid addition on quality, polyphenolic compounds and antioxidant capacity of cloudy apple juices. Eur Food Res Technol.

[34] Kurutas EB (2016). The importance of antioxidants which play the role in cellular response against oxidative/nitrosative stress: current state. Nutr J.

[35] Fuchs G, Rupprecht H (1983). Interactions of phenolic compounds with polyethylene glycols. Colloids Surf.

[36] Seker A, Arslan B, Chen S (2019). Recovery of polyphenols from grape pomace using polyethylene glycol (PEG)-grafted silica particles and PEG-assisted cosolvent elution. Molecules.

[37] Rhouma A, Chettaoui M, Krid S, Elbsir H, Msallem M, Triki MA (2013). Evaluation of susceptibility of an olive progeny (Picholine × Meski) to olive leaf spot disease caused by *Fusicladium oleagineum*. Eur J Plant Pathol.

[38] Koochak H, Puthiyaveetil S, Mullendore DL, Li M, Kirchhoff H (2019). The structural and functional domains of plant thylakoid membranes. Plant J.

[39] Chazaux M, Schiphorst C, Lazzari G, Caffarri S (2022). Precise estimation of chlorophyll a, b and carotenoid content by deconvolution of the absorption spectrum and new simultaneous equations for Chl determination. Plant J.

[40] Canino G, Ros F, Bassi R (2002). Chromophore organization in the higher-plant photosystem II antenna protein CP26. Biochemistry.

[41] Mustapa AN, Martin A, Gallego JR, Mato RB, Cocero MJ (2015). Microwave-assisted extraction of polyphenols from *Clinacanthus nutans* Lindau medicinal plant: energy perspective and kinetics modeling. Chem Eng Process Process Intensif.

[42] Ainsworth EA, Gillespie KM (2007). Estimation of total phenolic content and other oxidation substrates in plant tissues using Folin-Ciocalteu reagent. Nat Protoc.

[43] Nja RB, Merceron B, Faucher M, Fleurat-Lessard P, Béré E (2018). NaCl—changes stem morphology, anatomy and phloem structure in lucerne (*Medicago sativa* Cv. Gabès): comparaison of upper and lower internodes. Micron.

[44] Rantala M, Tikkanen M, Aro E-M (2017). Proteomic characterization of hierarchical megacomplex formation in *Arabidopsis* thylakoid membrane. Plant J.

[45] Offor BC, Mhlongo MI, Steenkamp PA, Dubery IA, Piater LA (2022). Untargeted metabolomics profiling of *Arabidopsis* WT, lbr-2-2, and bak1-4 mutants following treatment with two LPS chemotypes. Metabolites.

[46] Tchoumtchoua J, Mathiron D, Pontarin N, Gagneul D, Van Bohemen A-I, Otogo N’Nang E (2019). Phenolic profiling of flax highlights contrasting patterns in winter and spring varieties. Molecules.

[47] García-Villalba R, Espín JC, Tomás-Barberán FA, Rocha-Guzmán NE (2017). Comprehensive characterization by LC-DAD-MS/MS of the phenolic composition of seven *Quercus* leaf teas. J Food Compos Anal.

[48] Farag MA, Ezzat SM, Salama MM, Tadros MG (2016). Anti-acetylcholinesterase potential and metabolome classification of 4 *Ocimum* species as determined via UPLC/qTOF/MS and chemometric tools. J Pharm Biomed Anal.

