# Supplementary material for: An efficient protocol for extracting thylakoid membranes and total leaf proteins from Posidonia oceanica and other polyphenol-rich plants
Source: Plant Methods. 2024 Mar 11;20:38. doi: 10.1186/s13007-024-01166-7 (PMC10929114; doi:10.1186/s13007-024-01166-7)
Supplement: Supplementary file 2 — Additional file 2: Figure S1. Chlorophyll extraction yield using conventional and PVC protocols from selected plant species. Figure S2. 2D Urea-PAGE for the identification of P. oceanica supercomplexes isolated by CN-PAGE. Figure S3. Oxygen production rate from A. thaliana thylakoids (PVC protocol) and P. oceanica (2 m) thylakoids (conventional protocol, conventional protocol + 5% VitC and PVC protocol). Figure S4. 2D Urea-PAGE for the identification of peptides of supercomplexes isolated by BN-PAGE from various plant species. Figure S5. Organization and ultrastructure of leaf tissues from A. thaliana and Q. pubescens. Figure S6. Leaf chlorophyll content from various plant species. [file 13007_2024_1166_MOESM2_ESM.pdf]

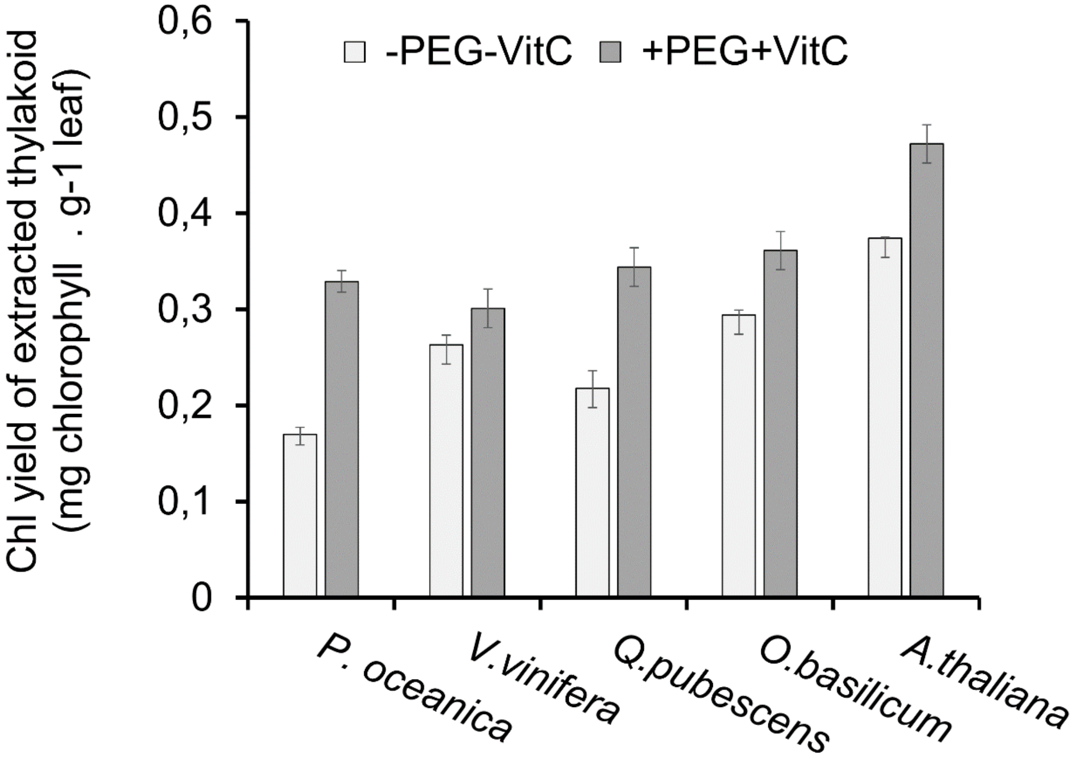

Figure S1: Chlorophyll extraction yield using conventional and PVC protocols from selected plant species.

The yield is based on the total chlorophyll mass (mg) of the isolated thylakoid preparation (measured in acetone) normalized by the initial fresh mass of plant material introduced in the blender. Raw data and calculations are presented in **Additional file 1: Data S2.** Data represents the mean of two replicates + SD.

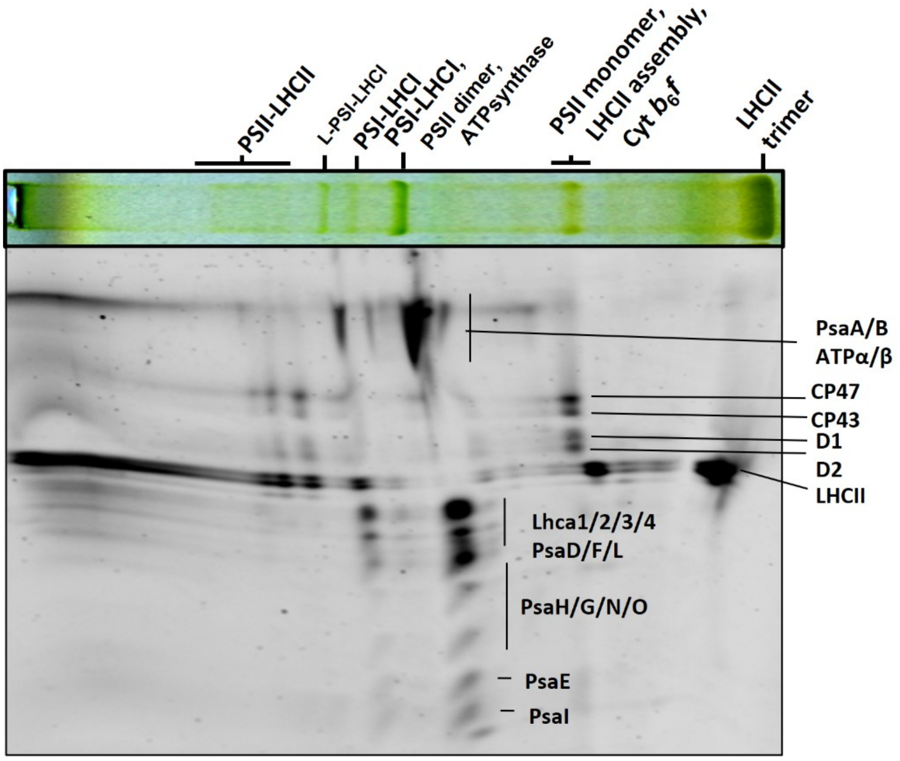

Figure S2: 2D Urea-PAGE for the identification of *P. oceanica* supercomplexes isolated by CN-PAGE. The lane of CN-PAGE from Figure 5B (5% PEG) was incubated in the denaturing Laemmli buffer and loaded on top of a 13% polyacrylamide gel. After electrophoresis, the gel was stained with Sypro™Ruby. The peptide assignment is based on [1] and [44].

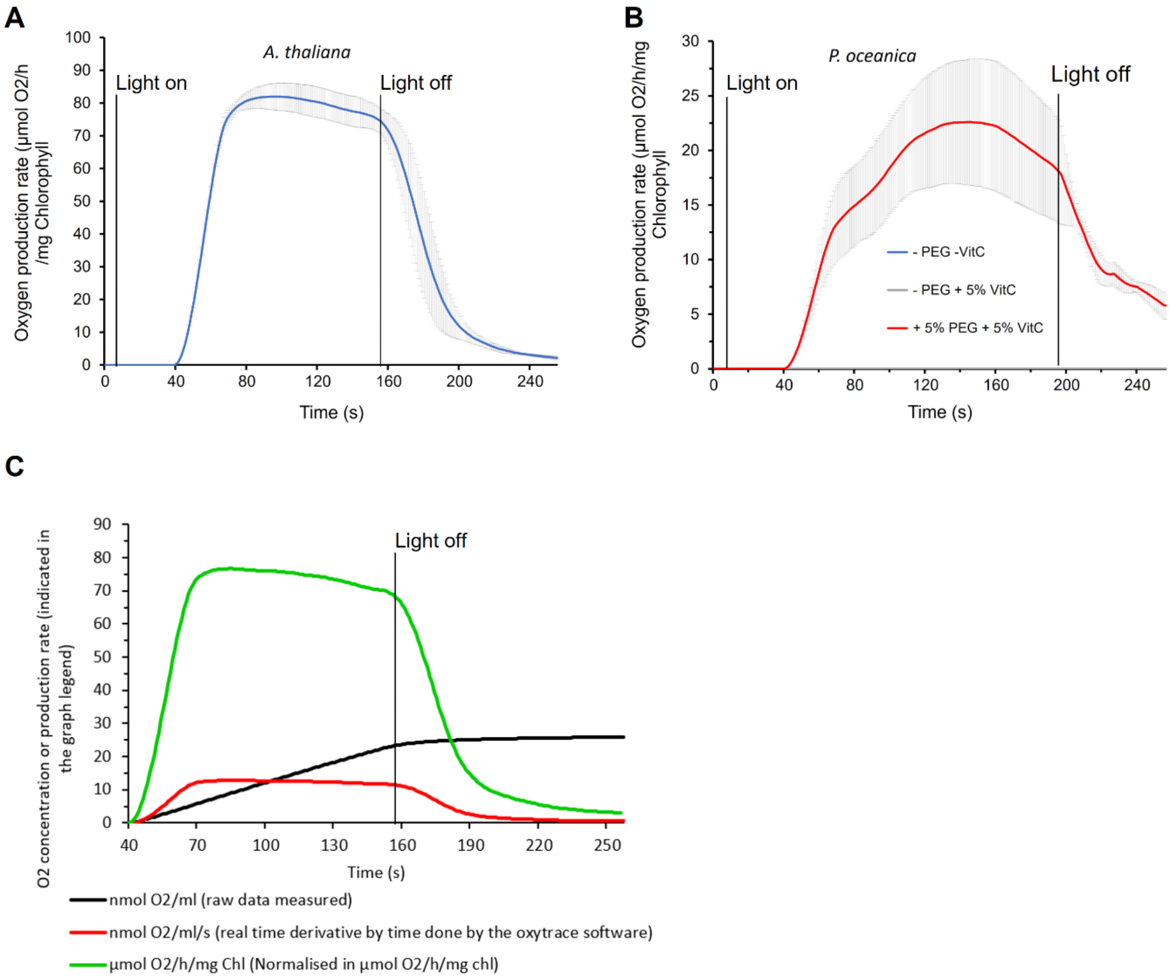

Figure S3: Oxygen production rate from *A. thaliana* thylakoids (PVC protocol) and *P. oceanica* (2m) thylakoids (conventional protocol, conventional protocol + 5% VitC and PVC protocol.). Thylakoids were illuminated at 1500 µmol photon/m²/s. Thylakoids were diluted at 10 µg/ml chlorophyll. Additionally, 50 mM HCO_3^- was added to *P. oceanica* thylakoid preparations.

a: Oxygen production rate (OPR) of *A. thaliana* thylakoids normalized by the quantity of Chlorophyll. **b**: OPR of *P. oceanica* thylakoids normalized by the quantity of Chlorophyll. All data are shown as the mean of technical triplicate + standard deviation. **c**: Representation of the raw data and normalized oxygen production rate measurements in *A. thaliana.* On the same graph is represented the raw data measured by the Clark electrode monitored by the oxytrace+ software (nmol O2/mL), the "raw" derivative calculated by the software which expresses the rate of O2 production in nmol/mL/s, and the normalized derivative in µmol O2/h/mg chlorophyll.

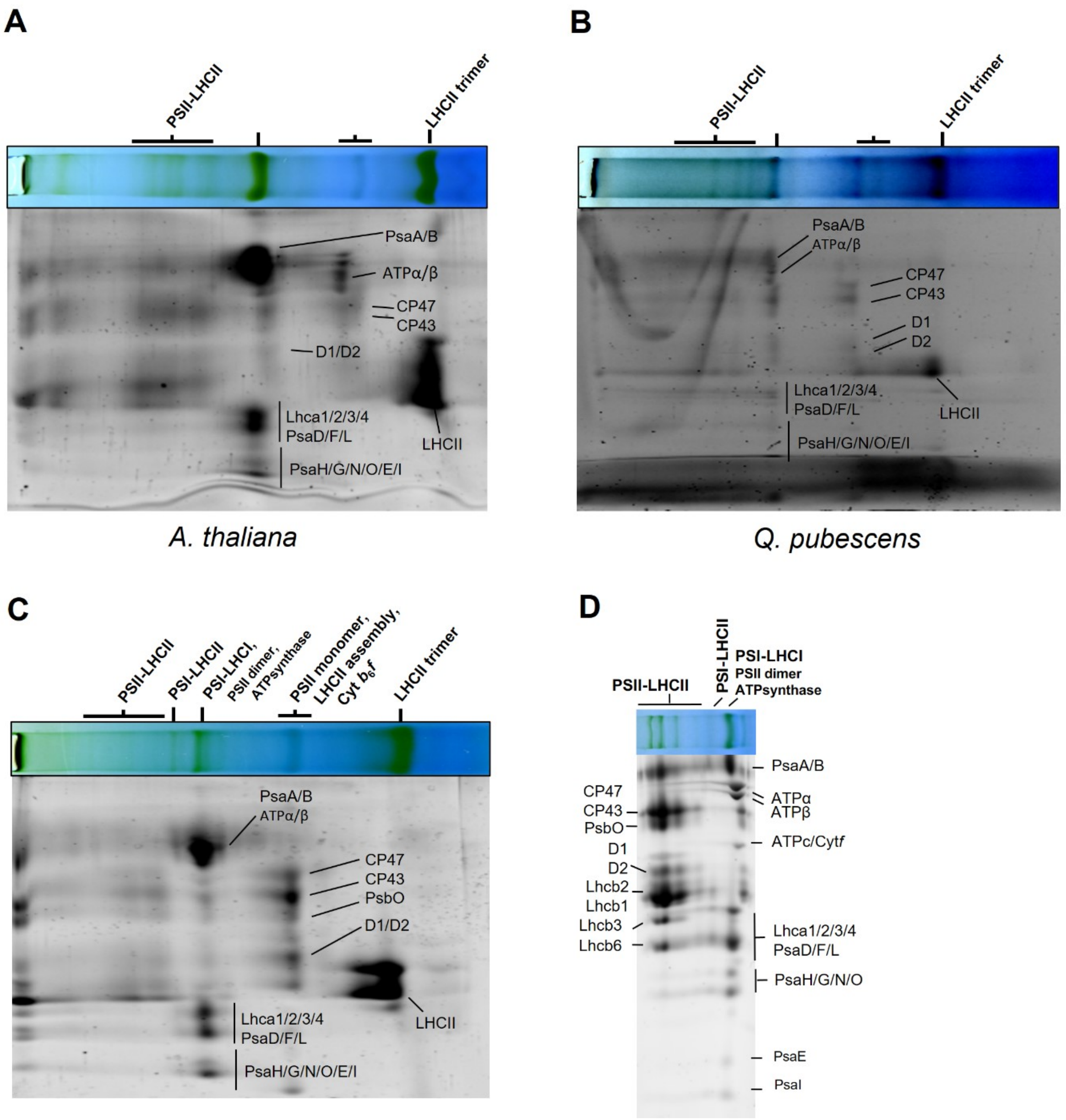

Figure S4: 2D Urea-PAGE for the identification of peptides of supercomplexes isolated by BN-PAGE from various plant species.

The lanes of BN-PAGE presenting the best separation and resolution from Figure 6 (+PEG, +VitC and 2%DDM) were incubated in Laemmli buffer and loaded on the top of a 13% polyacrylamide gel. After electrophoresis, the gel was stained with Sypro™Ruby. The peptide assignment is based on [1] and [44]. **(A)** *A. thaliana*, **(B)** *Q. pubescens*, **(C)** *V. vinifera*, **(D)** *O. basilicum*.

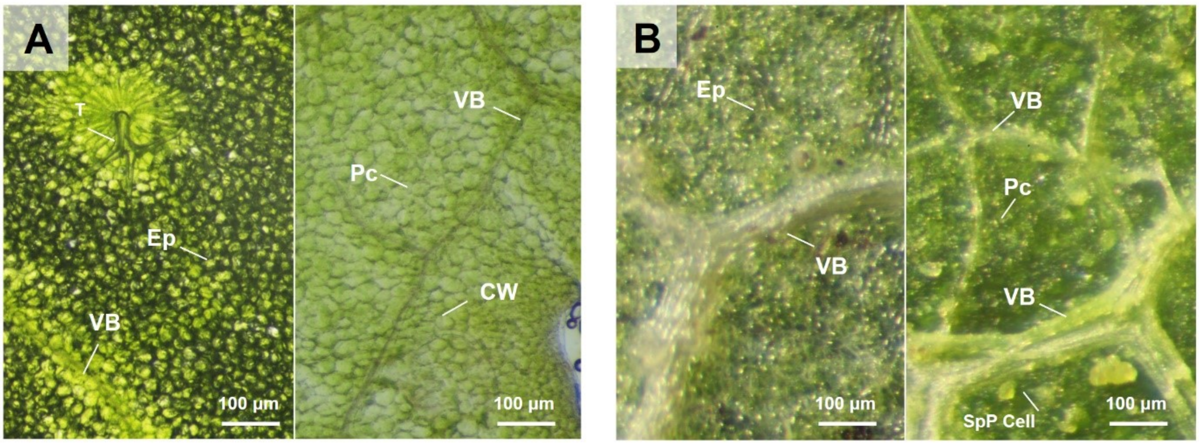

Figure S5: Organization and ultrastructure of leaf tissues from *A. thaliana* and *Q. pubescens*.
Low magnification microscopy photographs of leaf tissue ultrastructure from *A. thaliana* (**A**) and *Q. pubescens* (**B**). Left: View of the abaxial epidermis. Right: The abaxial epidermis was removed to expose underlying parenchyma. CW: Cell wall, Ep: Epidermis, Pc: Parenchyma, SpP cell: Spongy parenchyma cell, VB: Vascular bundle.

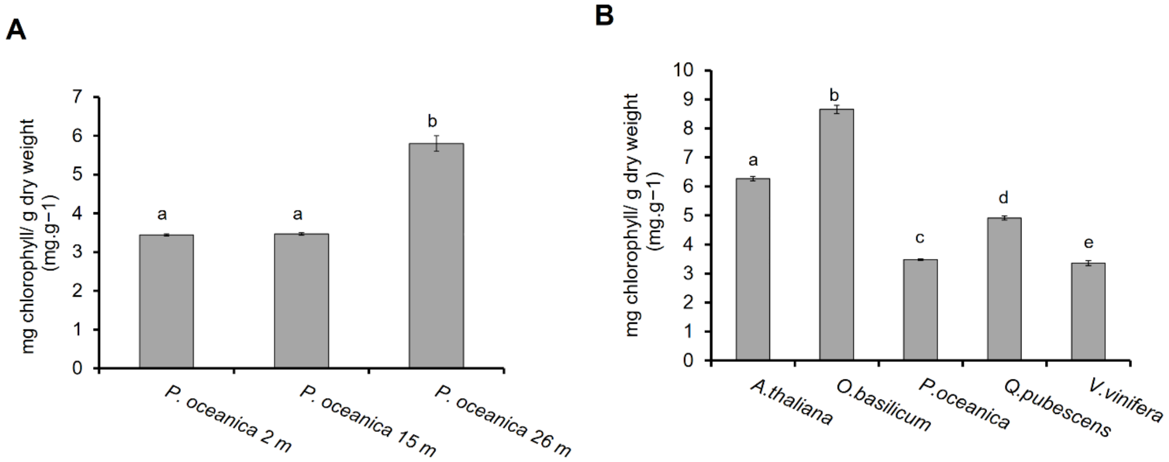

Figure S6: Leaf chlorophyll content from various plant species.
(A) Chlorophyll content of leaves from *P. oceanica* from the 3 tested depths. **(B)** Chlorophyll content of leaves from various species, including *P. oceanica* from 15 m depth. The chlorophyll content from leaves was normalized by the mass of freeze-dried material. Data are represented as the mean of four replicates + SD. Different letters indicate significant differences at $P < 0.05$, as determined using the Shapiro-Wilk normality test followed by the student's t-distribution analysis. Data are presented in **Additional file 1: Data S1**.
