# Supplementary material for: An efficient protocol for extracting thylakoid membranes and total leaf proteins from Posidonia oceanica and other polyphenol-rich plants
Source: Plant Methods. 2024 Mar 11;20:38. doi: 10.1186/s13007-024-01166-7 (PMC10929114; doi:10.1186/s13007-024-01166-7)
Supplement: Supplementary file 3 — Additional file 3: Method S1. Detailed protocol for the extraction of the thylakoid membranes from P. oceanica and various plant species. [file 13007_2024_1166_MOESM3_ESM.pdf]

Supplementary method S1: Detailed protocol for the extraction of the thylakoid membranes from *P. oceanica* and various plant species.

Buffer preparation and composition:

- Thylakoid membranes were extracted by grinding leaves in two distinct grinding buffers (GB).

For the conventional protocol, the buffer was composed of 20 mM Tricine pH 7.8, 0.3 M sorbitol, 10 mM EDTA, 10 mM $NaHCO_3$, 0.15% beef serum albumin (BSA), 10 mM of phosphatase inhibitor NaF, 5 mM benzamidine, 5 mM caproic acid.

For the PVC protocol, the grinding buffer described above was supplemented with 5% Polyethylene glycol 4000 (PEG4000) and 5% ascorbic acid (Vitamin C, Sigma).

- Chloroplasts were burst using a hypotonic buffer (25 mM Hepes-KOH pH 7.5, 25 mM sorbitol, 5 mM NaCl, 5 mM $MgCl_2$, 5 mM KCl, 10 mM NaF, and 5 mM Benzamidine, 5 mM caproic acid, cOmplete™ Protease Inhibitor Cocktail).
- The extracted thylakoid membranes were stored in the storage buffer (50 mM Hepes pH 7.5, 0.3 M sorbitol, 10 mM NaCl, 5 mM $MgCl_2$, 10 mM NaF, cOmplete™ Protease Inhibitor Cocktail).

Note 1: Different PEG MW (1000-4000-8000-10000) were tested but no significant difference was observed (data not shown). We thereby conserved PEG 4000 as used for our preliminary assay.

Note 2: Vitamin C was prepared as a stock solution of 25% at pH 7.0 and degassed to avoid oxidation of ascorbic acid. The buffer was stored at 4°C in the dark. Sorbitol was prepared as a 3 M stock solution and stored at 4°C.

Note 3: PEG powder was first dissolved in water (40% of the final volume of GB). Vitamin C and sorbitol were then added and preparation was completed with water up to 80% of the final volume of GB. All other compounds were then added. Finally, the grinding buffer pH was adjusted to 7.8, then completed with water to the final volume. Benzamidine, caproic acid, and protease inhibitor cocktail were added just before the use of the buffer.

Thylakoid membranes extraction:

The frozen leaf material (20 g) was homogenized in 500 mL of ice-cold GB (5 times 5 sec on, 5 sec off in a four-blade one-liter stainless blender tank). The leaf extract was then filtered on nylon tissue (20-25 µm pore) to remove unbroken cells and material. After centrifugation at 2500 *g* for 15 min at 4°C, the cell extract pellet was resuspended in 40-50 mL of hypotonic buffer to burst chloroplasts. The mixture was then transferred in a centrifuge tube and spun at 50-100 *g* for 2 min at 4°C to remove large particles and intact cells, which formed a loose pellet. The supernatant was then carefully transferred to a new tube and centrifuged at 3500 *g* for 10 min at 4°C. After centrifugation, the pellet was resuspended in a minimal volume of storage buffer (1-3 mL). Of note, the high-speed pellets obtained after treatment with the hypotonic buffer could be composed of different layers, the upper phase being dark green and containing the photosynthetic membranes. Thus, only the upper green layer was carefully resuspended with a thin brush to avoid incorporation of the undesired pelleted residues.
