# Supplementary material for: An efficient protocol for extracting thylakoid membranes and total leaf proteins from Posidonia oceanica and other polyphenol-rich plants
Source: Plant Methods. 2024 Mar 11;20:38. doi: 10.1186/s13007-024-01166-7 (PMC10929114; doi:10.1186/s13007-024-01166-7)
Supplement: Supplementary file 4 — Additional file 4. [file 13007_2024_1166_MOESM4_ESM.pdf]

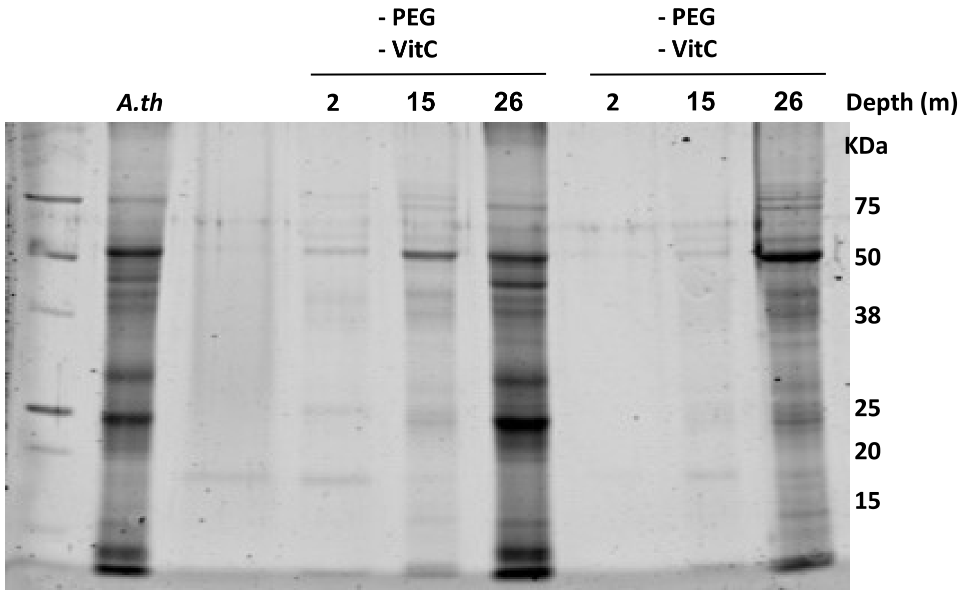

Figure 1B

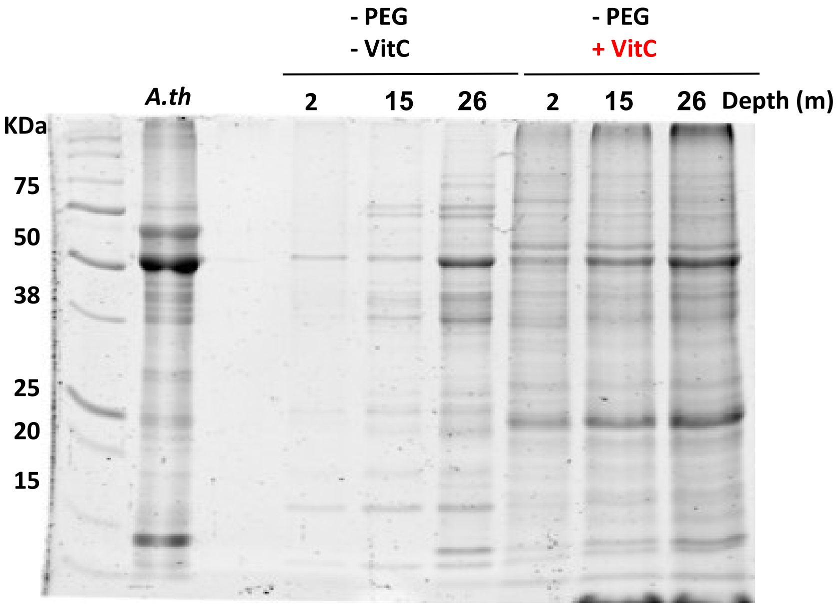

Figure 4A

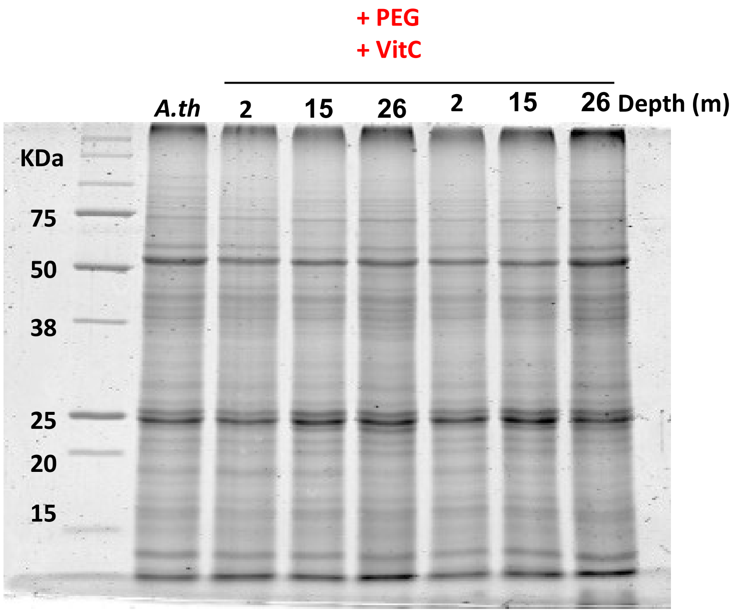

Figure 4B

Figure 4

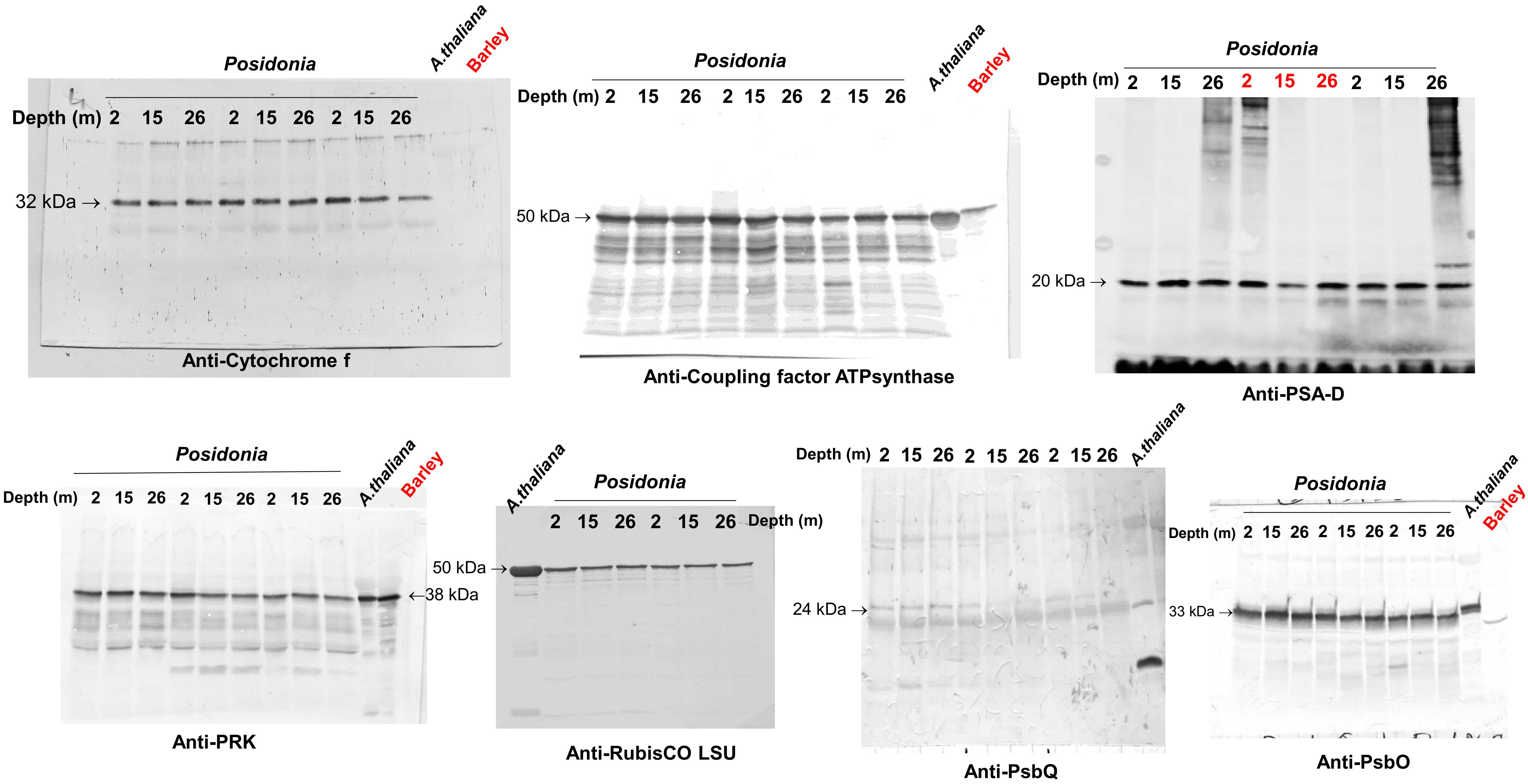
Posidonia
A.thaliana
Barley
Depth (m) 2 15 26 2 15 26 2 15 26
32 kDa →
Anti-Cytochrome f
Posidonia
A.thaliana
Barley
Depth (m) 2 15 26 2 15 26 2 15 26
50 kDa →
Anti-Coupling factor ATPsynthase
Posidonia
Depth (m) 2 15 26 2 15 26 2 15 26
20 kDa →
Anti-PSA-D
Posidonia
A.thaliana
Barley
Depth (m) 2 15 26 2 15 26 2 15 26
←38 kDa
Anti-PRK
A.thaliana
Posidonia
2 15 26 2 15 26 Depth (m)
50 kDa →
Anti-RubisCO LSU
Depth (m) 2 15 26 2 15 26 2 15 26
A.thaliana
24 kDa →
Anti-PsbQ
Posidonia
A.thaliana
Barley
Depth (m) 2 15 26 2 15 26 2 15 26
33 kDa →
Anti-PsbO

Figure 5A

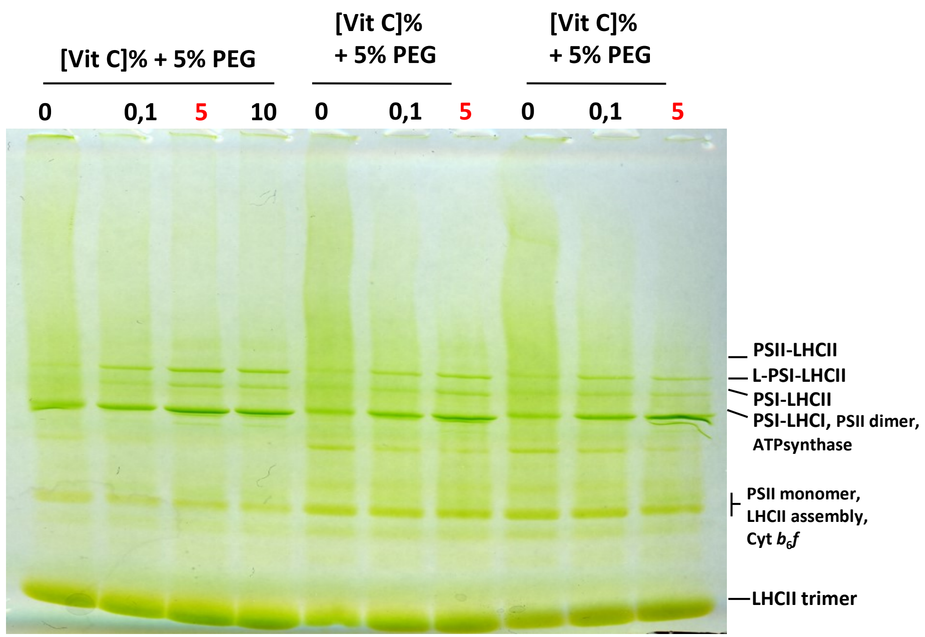

Figure 5B

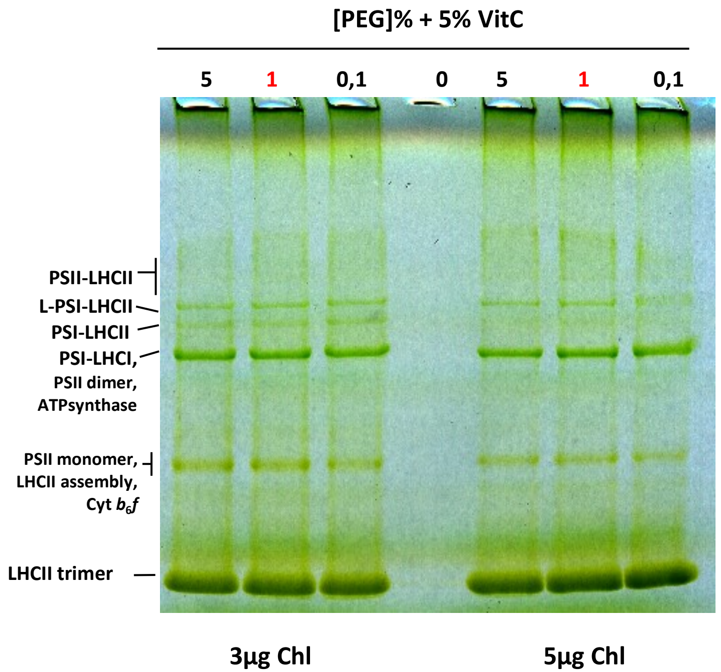

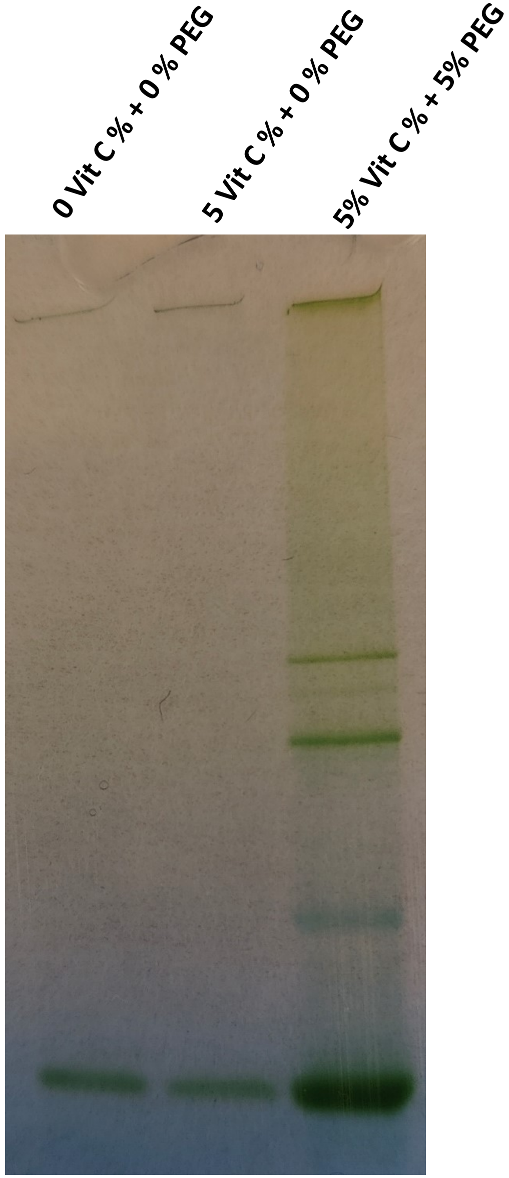

Figure 1A and 3A-3B

Figure 6

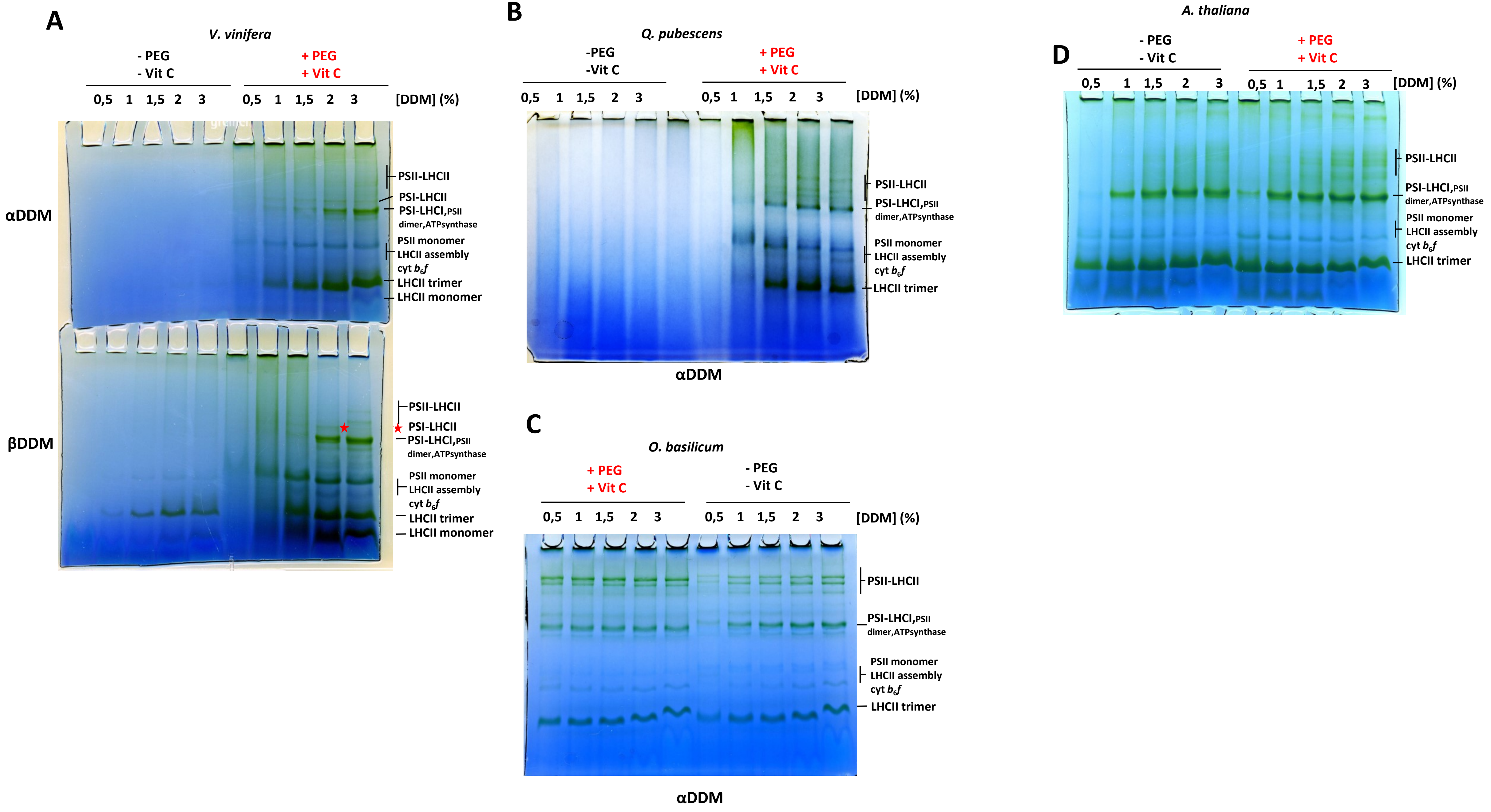
A
V. vinifera
- PEG
- Vit C
+ PEG
+ Vit C
0,5 1 1,5 2 3 0,5 1 1,5 2 3 [DDM] (%)
αDDM
PSII-LHCII
PSI-LHCII
PSI-LHCI,PSII dimer,ATPsynthase
PSII monomer
LHCII assembly
cyt b_6f
LHCII trimer
LHCII monomer
βDDM
PSII-LHCII
PSI-LHCII
PSI-LHCI,PSII dimer,ATPsynthase
PSII monomer
LHCII assembly
cyt b_6f
LHCII trimer
LHCII monomer
B
Q. pubescens
-PEG
-Vit C
+ PEG
+ Vit C
0,5 1 1,5 2 3 0,5 1 1,5 2 3 [DDM] (%)
PSII-LHCII
PSI-LHCI,PSII dimer,ATPsynthase
PSII monomer
LHCII assembly
cyt b_6f
LHCII trimer
αDDM
C
O. basilicum
+ PEG
+ Vit C
- PEG
- Vit C
0,5 1 1,5 2 3 0,5 1 1,5 2 3 [DDM] (%)
PSII-LHCII
PSI-LHCI,PSII dimer,ATPsynthase
PSII monomer
LHCII assembly
cyt b_6f
LHCII trimer
αDDM
A. thaliana
D
- PEG
- Vit C
+ PEG
+ Vit C
0,5 1 1,5 2 3 0,5 1 1,5 2 3 [DDM] (%)
PSII-LHCII
PSI-LHCI,PSII dimer,ATPsynthase
PSII monomer
LHCII assembly
cyt b_6f
LHCII trimer

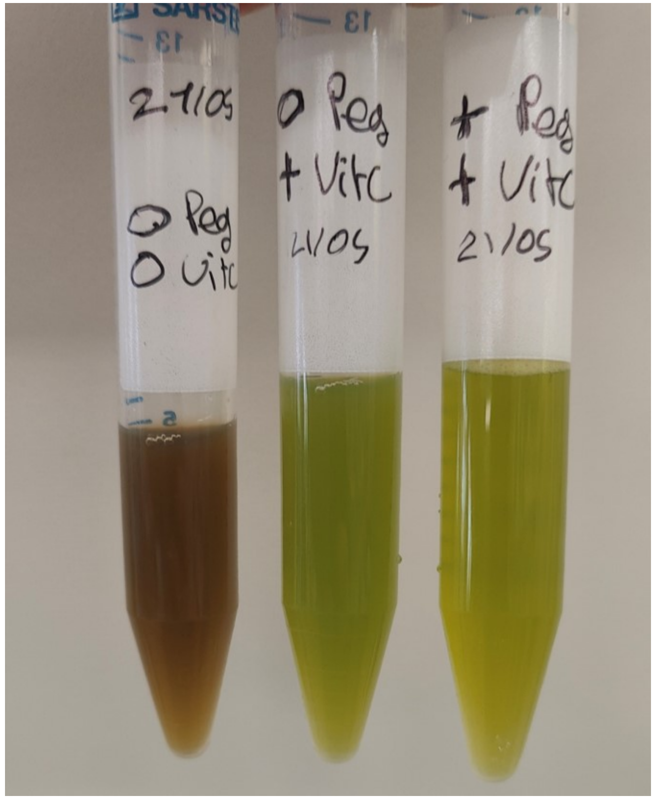

Filtrat

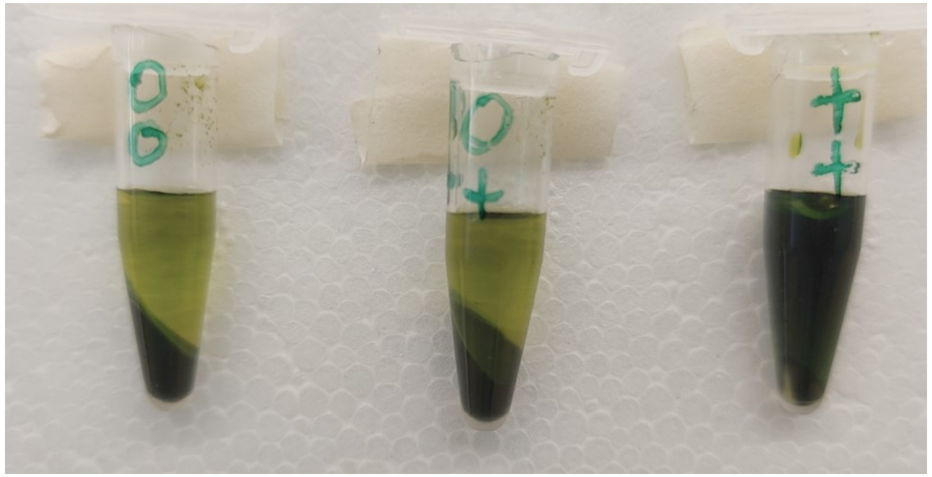

Centrifugation pellet

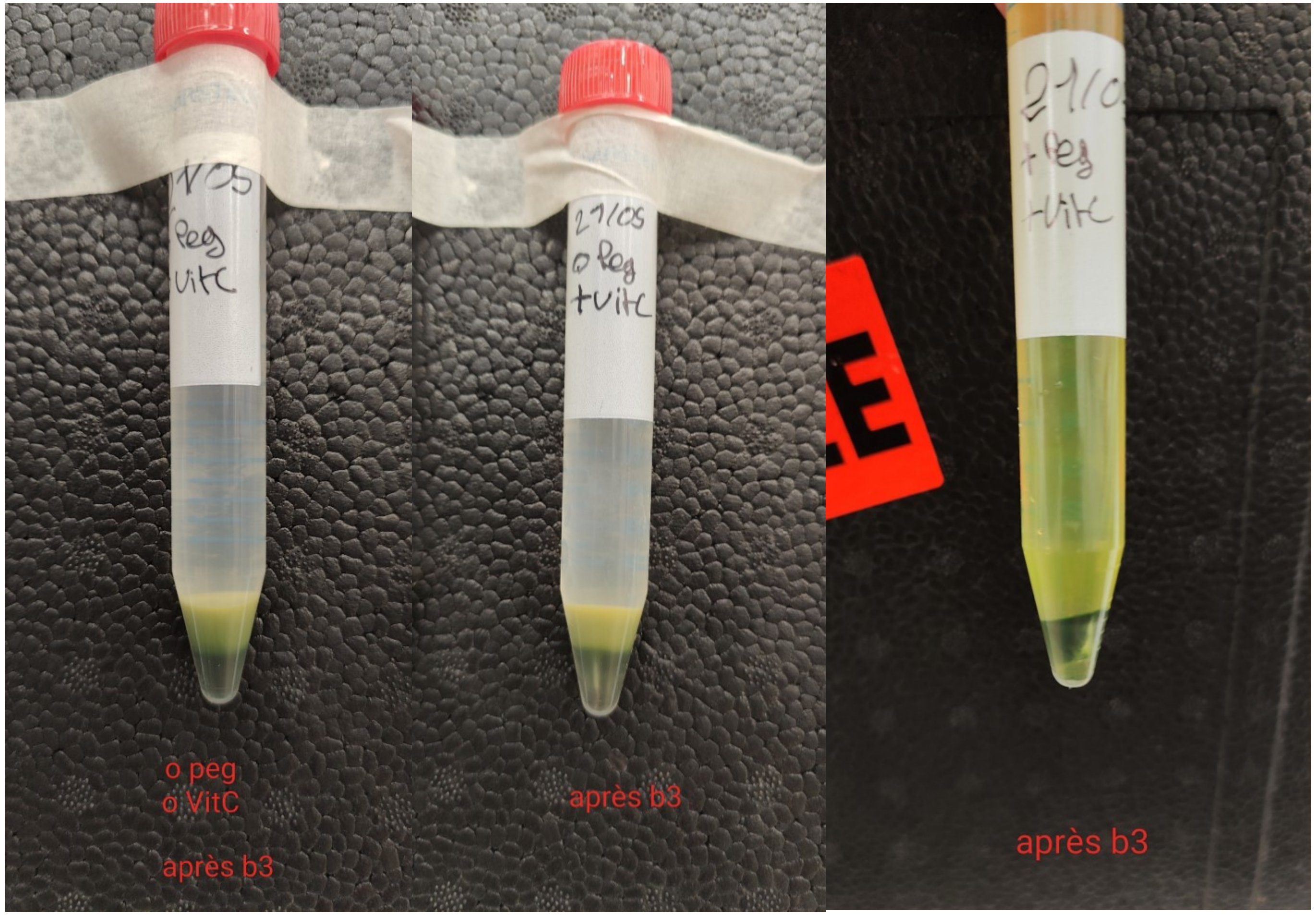

Centrifugation pellet
